# Optical Processes behind Plasmonic Applications

**DOI:** 10.3390/nano13071270

**Published:** 2023-04-03

**Authors:** Viktoriia E. Babicheva

**Affiliations:** Department of Electrical and Computer Engineering, University of New Mexico, Albuquerque, NM 87106, USA; vbb@unm.edu

**Keywords:** nanoparticles, metasurfaces, nanoparticle array, photovoltaics, light sources, sensors, biosensors, surface plasmon polaritons, localized surface plasmon resonances, collective resonances, 2D materials, bound state in the continuum

## Abstract

Plasmonics is a revolutionary concept in nanophotonics that combines the properties of both photonics and electronics by confining light energy to a nanometer-scale oscillating field of free electrons, known as a surface plasmon. Generation, processing, routing, and amplification of optical signals at the nanoscale hold promise for optical communications, biophotonics, sensing, chemistry, and medical applications. Surface plasmons manifest themselves as confined oscillations, allowing for optical nanoantennas, ultra-compact optical detectors, state-of-the-art sensors, data storage, and energy harvesting designs. Surface plasmons facilitate both resonant characteristics of nanostructures and guiding and controlling light at the nanoscale. Plasmonics and metamaterials enable the advancement of many photonic designs with unparalleled capabilities, including subwavelength waveguides, optical nanoresonators, super- and hyper-lenses, and light concentrators. Alternative plasmonic materials have been developed to be incorporated in the nanostructures for low losses and controlled optical characteristics along with semiconductor-process compatibility. This review describes optical processes behind a range of plasmonic applications. It pays special attention to the topics of field enhancement and collective effects in nanostructures. The advances in these research topics are expected to transform the domain of nanoscale photonics, optical metamaterials, and their various applications.

## 1. Introduction

Plasmonic applications are based on the optical processes that occur in nanostructures incorporating materials with negative permittivity and free-career oscillations. These processes involve coupling of light and the collective oscillation of electrons (known as plasmons) at the metal–dielectric interface. The size, shape, and composition of the nanostructures can be engineered to manipulate the properties of the plasmons, such as their resonance frequency and localization, which, in turn, can enable a wide range of applications in various fields [1,2,3,4].

**Figure 1:**
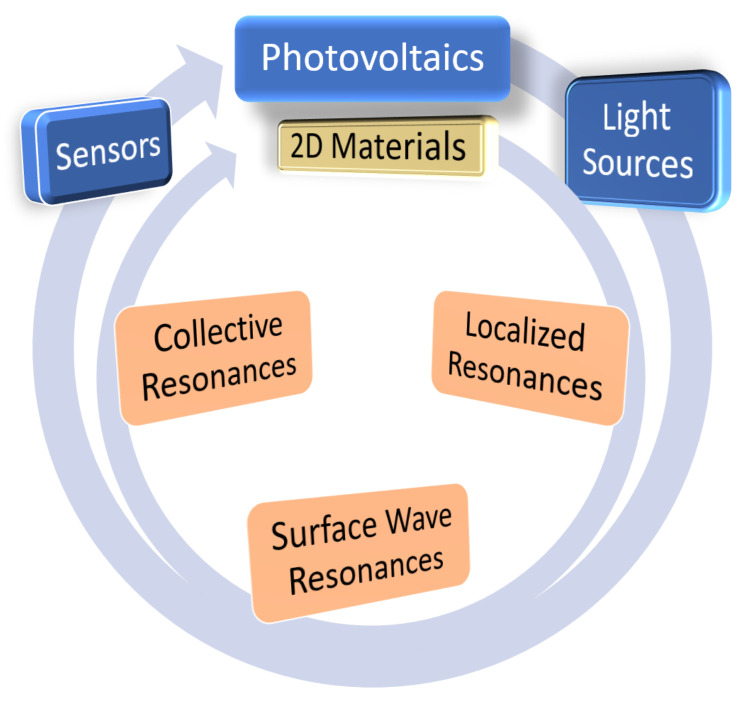
Plasmonic applications, devices, and sensors have gained widespread use in various fields, including biology, chemistry, and environmental monitoring. This review explores a range of plasmonic phenomena, including localized nanoparticle and propagating surface wave resonances, as well as processes in two-dimensional materials and collective multipolar excitations. In addition, this review examines plasmonic applications in enhancing photovoltaic efficiency, creating advanced light sources, and developing better sensors.

Tuning the properties of plasmonic nanostructures can facilitate the development of plasmonic applications by enabling control of light-matter interactions at the nanoscale. Plasmonics offers exciting possibilities for the creation, manipulation, and detection of signals through various processes, including generation, processing, transmission [1], and sensing [5]. All of these processes can occur at optical frequencies and potentially can find applications in many fields, including optical communications, biophotonics, sensing, chemistry, and medicine (Figure 1).

Plasmonic nanostructures, including those that support localized surface plasmon resonance, possess high electromagnetic fields that can be harnessed for a variety of applications. Localized surface plasmon resonance can lead to a strong enhancement of local electromagnetic fields, enhancing the functionality of various applications, such as sensing and spectroscopy. Another important process is surface-enhanced Raman scattering (SERS), which involves the interaction between incident light, plasmons, and molecules adsorbed on the surface of plasmonic nanostructures, resulting in a dramatic enhancement of the Raman signal. Surface-enhanced Raman scattering has important applications in analytical chemistry, biosensing, and imaging.

Gold nanoparticles can be made more effective in killing pathogens when they are combined with certain chemical compounds and exposed to near-infrared light. Both in vitro and in vivo studies have shown that gold-based nanocoatings can efficiently kill pathogens when exposed to near-infrared light. Additionally, the use of polymer nanocoatings on gold nanorods has shown promise in eradicating bacteria, particularly biofilms, when exposed to near-infrared light [4,6].

Scattering and interference of waves in plasmonic nanostructures can be exploited for improved performance in various applications. They often rely on other optical processes, such as plasmon hybridization, which involves the coupling between plasmons in different nanostructures, and Fano resonance, which arises from the interference between a discrete resonance and broad background continuum. These processes can be used to achieve better performance for various applications, such as plasmon-enhanced light harvesting in photovoltaics, plasmon-mediated energy transfer in nanoscale devices, and plasmon-induced hot electron generation in catalysis and photocatalysis.

The distinctive optical characteristics of plasmonic nanostructures render them attractive options for numerous applications in fields such as sensing, spectroscopy, and nanophotonics. Plasmonics is a booming research field aimed at understanding and practically applying the electromagnetic properties of nanostructured metals [2,7,8]. Plasmonic nanostructures offer the ability to manipulate and control optical fields at the nanoscale with great precision. These nanostructures can find use in diverse applications where increased efficiency or specificity is required. Resonant nanostructures provide much-needed strength in light-matter interaction, including significant enhancement of electromagnetic fields, their high localization, and large optical cross-sections of absorption and scattering processes.

The application of plasmonics has opened up a unique opportunity for nanoelectronics and nanophotonics to work in synergy, offering novel approaches in the designs of various applications. The field of plasmonics has long been seen as a promising area that can enable a unique integration of two major technologies, namely, nanoelectronics and nanophotonics. The crucial factor lies in the utilization of cost-effective planar fabrication processes, as well as the seamless integration with existing systems. This allows for novel approaches in the designs of applications, such as on-chip optics, “lab-on-a-chip" applications in biology and medicine, quantum data storage, and information processing. Advances in plasmonics include the enhancement of electric fields in the proximity to metal surfaces, allowing the measurement of single (bio)molecule processes that otherwise would be invisible [9,10,11], the surface-plasmon-polaritons-mediated transfer of energy from donor to acceptor molecules more than 100 nm apart, the enhanced light emission from quantum wells, and the world’s most miniature lasers [12].

This review is divided into nine sections, each of which focuses on different aspects of plasmonic processes and their applications. The first part of the review covers the underlying processes of plasmonic applications. Section 2 includes localized resonances, enhanced optical fields, energy localization, and quantum plasmonic. Section 3 is dedicated to propagating plasmonic surface waves. Section 4 provides a brief description of two-dimensional materials and their inclusion in plasmonic nanostructures and their applications. In Section 5, the collective effects in nanoparticle arrays are discussed, and it is shown how the periodic arrangement of nanoparticles results in narrow resonances with higher quality factors. The review then shifts its focus to specific plasmonic applications. Some of the most interesting plasmonic applications have been selected to be discussed in this review, without attempting to cover all of them. Section 6 discusses photovoltaics, Section 7 discusses light sources, and Section 8 covers sensors, including biosensors and chemical sensors, in the context of plasmonics. Table 1 summarizes the applications covered in this review and related processes involved in enhancing the efficiency and functionality of these applications. A detailed explanation of the underlying mechanisms is provided for each application. Finally, the review concludes with a summary of the topics and conclusions in Section 9.

## 2. Enhanced Optical Fields and Energy Localization

Many optical processes behind plasmonic applications are linked to LSPR, which is the collective oscillation of electrons in metal nanoparticles that is excited by incident light. These surface plasmon resonances are localized, nonpropagating optical excitations where the potential energy of the electric field or interactions of the polarization charges in the near field exchanges with the kinetic energy of the metal conduction electrons. Thus, surface plasmon resonances are electromechanical oscillations that are not subject to the diffraction limit in contrast to photons, which are electromagnetic oscillations. These excitations exist in the nanovicinity of metal surfaces (metal–dielectric interfaces). Their enhanced fields are responsible for the remarkable fundamental properties and a multitude of widely used applications of nanoplasmonics.

In nanostructures with LSPR at the metal surface with permittivity $\varepsilon_{m}$ at frequency ω, the quality factor $Q_{\mathrm{LSPR}}$ indicates the number of plasmon oscillations that occur before the field decays. It is commonly defined as

(1) QLSPR≡ω∂Re[εm(ω)]/∂ω2Im[εm(ω)],

or approximately

(2) QLSPR=−Re[εm]Im[εm].

One can see from this expression that the LSPR quality factor $Q_{\mathrm{LSPR}}$ linearly depends on Re[εm].

Unlike dielectrics, metals have free carriers, and plasmons are the collective excitations of these free electrons with respect to a positively charged background. Fundamental physical properties determine both the spatial and temporal scales in plasmonics. There exists a hierarchy of spatial scales relevant to optical phenomena. Among them, the largest is the radiation wavelength λ ∼ 1 µm, reduced wavelength ƛ ∼ 100 nm, the electron mean free path in metals ∼40 nm, and the most important is the skin depth ∼25 nm for metals such as gold, silver, platinum, copper, etc. It is particularly important that the field penetrates metals to the skin depth because the plasmonic phenomena occur due to the interaction of metal electrons with the electric field of the incident optical radiation.

These elementary excitations-surface plasmons can be treated both classically (using classical electrodynamics) and quantum-mechanically (employing quantum electrodynamics). The classical surface plasmon field serves as a basis for quantizing the surface plasmons. A propagating electromagnetic wave field cannot be localized to a region less than half a wavelength. This is due to the energy exchange between the electric and magnetic field components, which occurs at a distance of a quarter wavelength. The fields of the surface plasmons are extended over the regions whose size is only determined by the scale of the nanostructure itself. The enhancement of optical magnetic fields of the surface plasmons is small and does not play a role in the surface plasmon dynamics, which is the underlying cause of the nanoscale localization and scaling with the nanoparticle size.

The plasmonic phenomena occur for nanoparticles whose characteristic dimensions are less than the skin depth. In such a case, the electromagnetic field of the incident wave completely fills the nanoparticle and drives electron oscillations in the metallic nanostructure. Nanoscale light localization in plasmonic structures is a phenomenon drastically different from conventional electromagnetic waves. Moreover, the case of localized surface plasmons differs from the surface plasmon polaritons, which are propagating electromagnetic waves at the metal surfaces, confined and localized in its proximity. The electrical charges, which appear primarily on the metal surfaces, interact with each other creating restoring forces. These forces can be considered instantaneous Coulomb forces because retardation can be neglected under the condition when the characteristic size is less than the skin depth. These restoring forces and the mechanical (effective) mass of the electrons form electromechanical oscillators, which are eigenmodes of the nanoplasmonic systems called (localized) surface plasmons.

**Figure 2:**
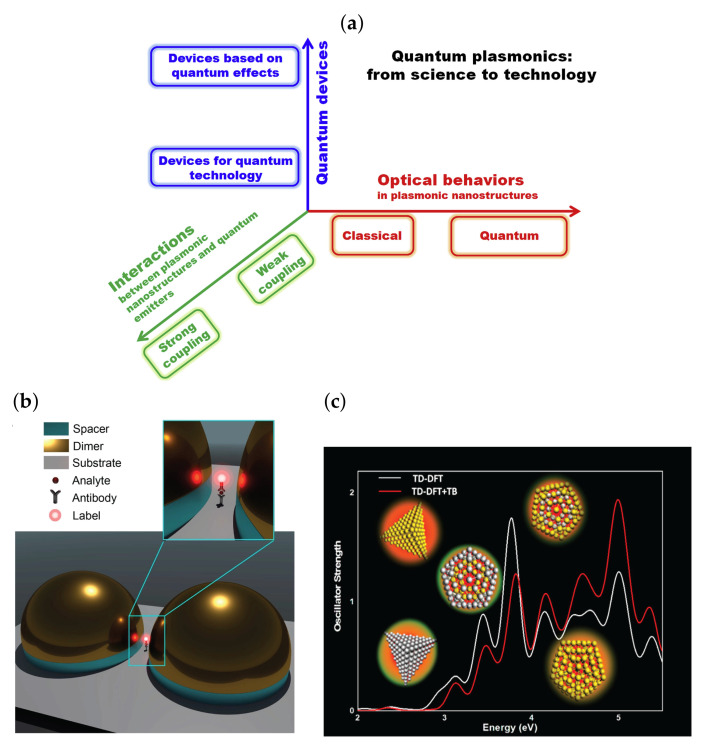
(**a**) Science and technology towards quantum plasmonic devices. (**b**) Plasmon-enhanced quantum immunoassay sensing. A schematic diagram of the strong-coupling immunoassay configuration shows that an immunoassay complex is trapped in the vicinity of a plasmonic hotspot within a nanodimer cavity made of gold hemispheres. (**c**) The plasmonic response of bimetallic assemblies with various proportions of silver and gold atoms obtained with an advanced density functional theory technique to calculate excited states. (**a**) Reproduced with permission from [13]. Copyright 2019 by Elsevier B.V. (**b**) Reproduced with permission from [17]. Copyright 2019 by American Chemical Society. (**c**) Reproduced from [20].

The ongoing miniaturization of plasmonic structures also gives rise to new theoretical challenges of fundamental nature (Figure 2a). Although nowadays plasmonic nanostructures are fabricated on deep subwavelength scales, the metal is still usually described in terms of a bulk local-response function. This response is dominated by the interaction of the free carriers with light, as described by the standard Drude response, which assumes a constant electronic density. However, the free carriers start to feel the metal surface as the structures become smaller, and as a consequence of this quantum confinement, the electron density becomes nonuniform in the proximity to the metal surface. Furthermore, the optical response becomes nonlocal (‘spatial dispersion’), which becomes important at distances ∼10 nm according to the latest insights. Quantum plasmonics aims to investigate the quantum nature of plasmons and their interactions with light, which could potentially lead to the development of new technologies in nanophotonics, sensing, and quantum information processing [13].

A recent exciting development is observing the breakdown of classical theory on individual metallic nanoparticles. Experimental studies on noble metallic nanostructures with nanometer-scale features have shown that the behavior of surface plasmon resonances differs from the predictions of the Drude model [14,15]. Energy electron loss spectroscopy (EELS) has been used to observe a significant blueshift of resonance energies as particle sizes decreased down to a few nanometers [16], whereas classical theory predicts no such shift at all. However, the observed blueshifts have been even significantly larger than expected. The prediction was based on a quantum confinement theory and hydrodynamic Drude theory, which estimates similar blueshifts but based on different concepts. Effects of the dielectric environment (substrates) have not been fully considered in the analysis of experiments. The challenge is, therefore, going beyond state-of-the-art and developing better analytical tools to determine which of the competing theories describe experiments best.

Plasmonic nanostructures offer a viable alternative to conventional bulky optical elements and devices in various applications. These nanostructures are ultra-thin, lightweight, and ultra-compact, making them capable of overcoming some of the limitations of their traditional counterparts. Over the last decade, scientists have extensively employed plasmonic nanostructures in classical-optics applications, and they have proven successful. In recent times, researchers have further extended the use of plasmonic nanostructures to the quantum realm.

The efficacy of a novel immunoassay detection method has recently been demonstrated by employing quantum emitters as sensing labels for the detection of antibody–antigen–antibody complexes [17]. Hemispherical nanoplasmonic open cavities have been utilized to facilitate room-temperature strong coupling (Figure 2b). The gaps between two plasmonic nanoparticles (e.g., dimer configuration) can lead to a significant enhancement of the electromagnetic field due to the LSPR supported by such nanostructure [3,18,19]. These gigantic field enhancements have important implications for surface-enhanced spectroscopies and sensing applications.

Recent work [17] has demonstrated the possibility for plasmonic experiments utilizing the quantum immunoassay sensing approach to ultimately attain the limit of single-analyte detection. Although this protocol involves quantum effects, i.e., strong coupling with a quantum emitter, to detect a classical object, such as an antigen, the technique has the potential to be extended to identify a quantum entity, such as an electronic spin found in nanodiamonds. The discovery of this new approach has unlocked the potential for plasmonics-based quantum sensing at room temperature.

The study of plasmonic properties in nanoparticles with a quantum mechanical approach, such as the time-dependent density functional theory with tight binding (TD-DFT+TB method), is another important approach in the field of quantum plasmonics [20]. The plasmonic features of pure silver, gold, and bimetallic silver-gold clusters have been studied by us using the TD-DFT+TB method that includes the molecular orbitals in the ground state calculated with a density functional theory and time-dependent tight-binding such as approximations of the coupling matrix utilized in the Casida’s linear response calculations (Figure 2c). The efficacy of this approach has been illustrated in accurately simulating the influence of alloying type, shape, and size on the plasmonic characteristics of tetrahedral and icosahedral elements, encompassing a range of approximately 3000 to 30,000 electronic states.

At the early stage of development, strongly fluctuating and enhanced local optical fields in metal fractal clusters were introduced [21,22,23,24]. It has been shown that these fields led to an enormous magnitude of SERS and established that this enhancement was significantly increased in the red and near-infrared parts of the spectrum [25]. Based on this theoretical prediction, single-molecule SERS was discovered [26]. Soon after, two intimately related and very important properties of the plasmonic near field were predicted: inhomogeneous localization and the formation of random nanospeckles of the local fields called the “hot spots” [27,28,29,30]. These hot spots are the most general way of the existence of nanoscale plasmonic fields, which have been confirmed in many experiments. This is related to the fact that the localization of optical energy on the nanoscale is not limited by any electromagnetic length: the electromagnetic scales are too large to determine the nanoplasmonic phenomena. On the other hand, the nonlocality radius, which also determines the onset of the Landau damping, is too small [31,32,33].

It has been established that the classical surface plasmon eigenmodes are inhomogeneously localized on all scales of the nanosystem from the maximum to the minimum [34]. The hot spots are highly visible because their strongest localization corresponds to the maximum localization of energy, which makes them very bright. Subsequently, a general theory of the deep subwavelength surface plasmon eigenmodes (also called quasi-static eigenmodes) has been developed. The surface plasmons that are strongly localized were also shown to be dark [35]. Though Anderson localization of surface plasmons does certainly exist for surface plasmons, the excitation and observation of the surface plasmon eigenmodes must necessarily be completed in the near field. Green’s function approach has been used based on the spectral expansion of the surface plasmon eigenmodes and the corresponding eigenvalues to describe many continuous-wave and ultra-fast nanoplasmonic phenomena [36,37,38,39,40,41].

## 3. Guiding and Confining Surface Plasmon Polaritons

On metal surfaces, light can couple to plasmons and form so-called surface plasmon polaritons (SPPs), which typically penetrate only ∼10–100 nm into the metal. Therefore, SPPs can be used to concentrate light in structures much smaller than the wavelength of light [1,42,43]. Metals are unsuited for guiding light further than ∼100 µm because of the energy dissipation and propagation losses. So traditionally, the optical guiding properties of metals were mainly studied as a probe to learn about the metal itself. A “holy grail" is to use SPPs to make nanoscale photonic circuits operating at optical frequencies. Further progress may bring together the fields of optics and electronics to the nanoscale characteristic sizes.

Integrated optical technologies provide significant advantages such as high bandwidth, low propagation loss, and noise reduction, but the diffraction-limited nature of light limits the minimum size of components, hindering the widespread adoption of integrated optical components. Plasmonic waveguides can provide a significant enhancement of the electromagnetic field due to the strong confinement of SPPs within the waveguide [44]. This field enhancement can be utilized to enhance the modulation efficiency of plasmonic modulators [45,46], which operate by changing the refractive index of the plasmonic waveguide through external stimuli such as electrical or optical signals. Hyperbolic metamaterials have been shown to greatly enhance the plasmonic field due to their unique anisotropic permittivity, which allows for the propagation of highly confined, subwavelength waves. The plasmonic field enhancement in hyperbolic metamaterials can be further enhanced by controlling the geometry and size of the hyperbolic-metamaterial structures, as well as by introducing defects or dopants to the material [47,48]. This enhanced plasmonic field in hyperbolic metamaterials has potential applications in areas such as sensing, light trapping, and nanophotonics, where highly localized fields are desirable.

The propagation constant $k_{\mathrm{SPP}}$ of surface plasmon polaritons confined to a dielectric-metal boundary is defined by a relatively simple analytical expression [49]. One can show that for this plasmonic waveguide with relatively low metal losses and its permittivity $\varepsilon_{m}$, the quality factor $Q_{\mathrm{SPP}}$ depends on Re[εm]2:

(3) QSPP≡Re[kSPP]2Im[kSPP]≈Re[εm]2εcIm[εm].

Here, $\varepsilon_{c}$ is the dielectric permittivity.

The propagation length *L* of SPP along the dielectric-metal interface excited with the external light with wavenumber $k_{0}$ is

(4) L=12Im[kSPP]≈Re[εm]2k0εc3/2Im[εm].

Assuming Re[kSPP]≈k0εc1/2 and considering the condition $\varepsilon_{c}<<\left|\varepsilon_{m}\right|$, Equation (4) yields the same power dependence on the real and imaginary parts of $\varepsilon_{m}$. Thus, the figure of merit (FoM) for the dielectric-metal waveguide can be defined as

(5) FoMdielectric-metal=Re[εm]2Im[εm].

For more complex waveguide designs, such as metal–dielectric–metal or dielectric–metal–dielectric, the powers of scaling law (*x* in $\varepsilon^{x}$ dependence) are not well defined. Waveguides need to be evaluated using various metrics, and the appropriate FoM should be selected based on the waveguide’s design [48]. In the case of long-range propagation in dielectric–metal–dielectric waveguides, the quality factor $Q_{\mathrm{LSPR}}$ is a more suitable metric for comparing plasmonic materials as constituent thin metal stripes. Furthermore, it is possible to determine the propagation length of various waveguides and establish empirical relationships for its dependence on the permittivity of either metal or hyperbolic metamaterials. However, conventional $Q_{\mathrm{SPP}}$ may not accurately reflect the propagation length of waveguides in the long-range regime. Therefore, other figures of merit must be utilized to compare metal or hyperbolic-metamaterial materials with varying real and imaginary components that need to be used in a specific waveguide design [48].

## 4. Plasmonic Nanostructures and Two-Dimensional Materials

Layered van der Waals materials have a unique band structure that results in strong light-matter interactions and excellent optoelectronic properties, including strong light absorption and emission, high carrier mobility, and a tunable bandgap [50,51]. The two-dimensional nature of transition metal dichalcogenides (TMDCs), such as disulfides and selenides of molybdenum and tungsten (MoS${}_{2}$, WS${}_{2}$, MoSe${}_{2}$, WSe${}_{2}$), enables the possibility of van der Waals heterostructures by stacking different layers on top of one another, which can lead to additional functionalities and applications. Transition metal dichalcogenides have shown great promise in a wide range of optoelectronic devices, including photodetectors, solar cells, light-emitting diodes, and transistors. The excellent potential application and performance of these materials in optoelectronic devices have been demonstrated experimentally. However, challenges still exist in the large-scale synthesis and integration of TMDCs into practical devices, as well as in the understanding and control of their electronic and optical properties, which require further research and development. The ultra-fast dynamics of metal plasmons induced by two-dimensional semiconductors in hybrid nanostructure arrays have been studied extensively, revealing novel physical phenomena and potential applications in optoelectronics and photonics [52].

**Figure 3:**
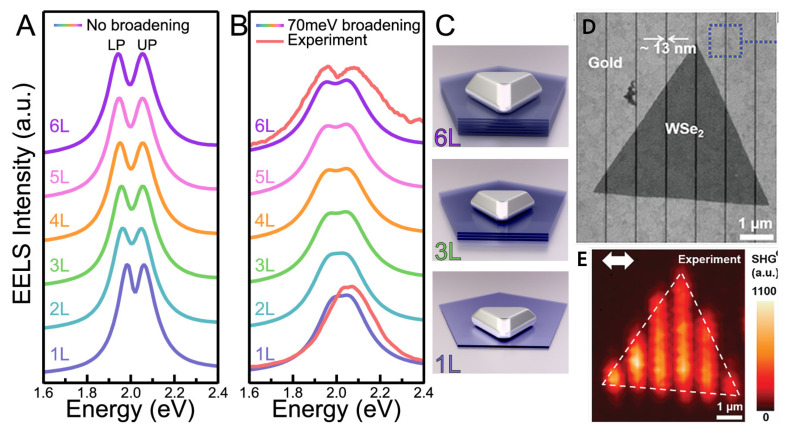
(**A**–**C**) The influence of WS${}_{2}$ thickness and EELS energy resolution on Rabi splitting. Electron energy loss spectra are calculated for a perfectly tuned coupled system consisting of a 75 nm Ag truncated nanopyramid on a WS${}_{2}$ flake on a 20 nm Si${}_{3}$N${}_{4}$ support, with WS${}_{2}$ thickness varying between one and six layers. The perfect adjustment is achieved by adjusting the height of each truncated nanopyramid while maintaining the length of the bottom face and the angles of the side face. Two scenarios are considered: (**A**) no spectral broadening and (**B**) an assumed energy resolution of 70 meV, which is the upper limit for 40–70 meV resolution experiments. Experimental electron energy loss spectra from coupled truncated nanopyramids on one and six layers of WS${}_{2}$ are compared to the red spectra in panel (**B**). (**C**) Schematic models of the zero-detuned condition for WS${}_{2}$ thicknesses of one, three, and six layers, with changes in truncated nanopyramid height to achieve perfect tuning. (**D**,**E**) Pump-laser-polarization-dependent second harmonic generation (SHG) mapping of monolayer WSe${}_{2}$ on gold trenches. (**D**) Image obtained from scanning electron microscopy (SEM) of a single-crystalline monolayer WSe${}_{2}$ flake on trenches having a spacing of 910 nm. (**E**) Experimental mappings of SHG obtained under resonant and nonresonant excitations for the same WSe${}_{2}$ flake located on the trenches shown in the SEM image. The white dashed lines delineate the edges of the WSe${}_{2}$ flake. The white arrows indicate the polarization directions of the pump laser, which are perpendicular to the trenches. The fabricated 150 nm-thick flat surface of monolayer WSe${}_{2}$ and sub-20 nm-wide gold trenches on flexible substrates demonstrated a ∼7000-fold enhanced SHG, without resonance broadening or background in the spectra, compared to WSe${}_{2}$ on as-grown sapphire substrates. (**A**–**C**) Reproduced with permission from [53]. Copyright 2019 by American Chemical Society. (**D**,**E**) Reproduced with permission from [54]. Copyright 2018 by American Chemical Society.

Polaritons are light-matter quasiparticles that have led to groundbreaking advancements in quantum optics and material science. Recently, researchers have successfully demonstrated the existence of plasmon-exciton polaritons (plexcitons) in hybrid materials that consist of metal nanoparticles and transition metal dichalcogenides. This has opened up exciting possibilities for realizing polaritonic effects in nanoscale systems at room temperature. Mapping the nanometer-scale characteristics of hybrid materials is challenging. One of the approaches is to use electron energy loss spectroscopy to map plexcitons spectroscopically in a hybrid system of plasmonic nanoparticles and few-layer two-dimensional materials with nanometer spatial resolution [53]. In the strong coupling regime, plexcitons hybridize and exhibit unexpected nanoscale variations that arise from their deep-subwavelength nature (Figure 3A–C). These findings offer new opportunities for studying polariton-related phenomena in transition metal dichalcogenides hybrid material systems at the local atomic structure level with high spatial resolution.

The nonlinear optical phenomena can be controlled by either the phase-matching condition, crystal symmetric, or both. Plasmonic nanostructures provide in-plane coupling that satisfies the phase-matching condition for two-dimensional materials, which conserves both momentum and energy simultaneously. The odd-order nonlinearity, for example, third harmonic generation, can be observed in systems with any symmetry, whereas even-order susceptibility vanishes in centrosymmetric material.

Due to their atomic thinness, two-dimensional materials typically have a short interaction length with the pump laser, leading to generally inefficient second-harmonic generation (SHG). Nevertheless, TMDCs with a lack of inversion symmetry on the surface can exhibit a pronounced SHG when subjected to a strong optical pump and plasmon-enabled light-matter interaction [54,55,56] (Figure 3D,E). Thus, two-dimensional monolayer TMDCs are promising materials for nanoscale nonlinear optical frequency conversion due to their large second-order nonlinear susceptibility and inversion asymmetry.

## 5. Narrow Collective Resonances

The theory of surface plasmon polariton had its roots in the early twentieth century when Robert Wood conducted his pioneering work in 1902 [57,58]. While studying metallic diffraction gratings, he observed two anomalies in his experimental data. Firstly, he noticed narrow bright and dark bands in the spectral response of the reflection of the metallic gratings, which depended on the incoming wavelength, the grating period, and the refractive indices of the surrounding media. Secondly, he observed that these phenomena occurred when the magnetic field vector of the incoming light was parallel to the grating grooves. Lord Rayleigh later explained these anomalies as manifestations of rapid variations in intensity.

In the subsequent research, a new theory explaining Wood’s anomalies was proposed, utilizing a guided wave approach rather than the conventional multiple scattering method [59]. This approach offers novel insights and a calculation method. It is demonstrated that two distinct types of anomalies may arise: (i) Rayleigh wavelength type, resulting from a new spectral order at a grazing angle, and (ii) resonance type, which is associated with the guided complex waves that the grating can support. A comprehensive theoretical framework was employed to account for the standing waves in the grating grooves providing detailed information on the locations and shapes of the anomalies. A specific example was utilized to obtain rigorous results, with an explicit determination of the amplitudes of all spectral orders. The effects of Wood’s anomalies were clearly demonstrated in a range of cases.

**Figure 4:**
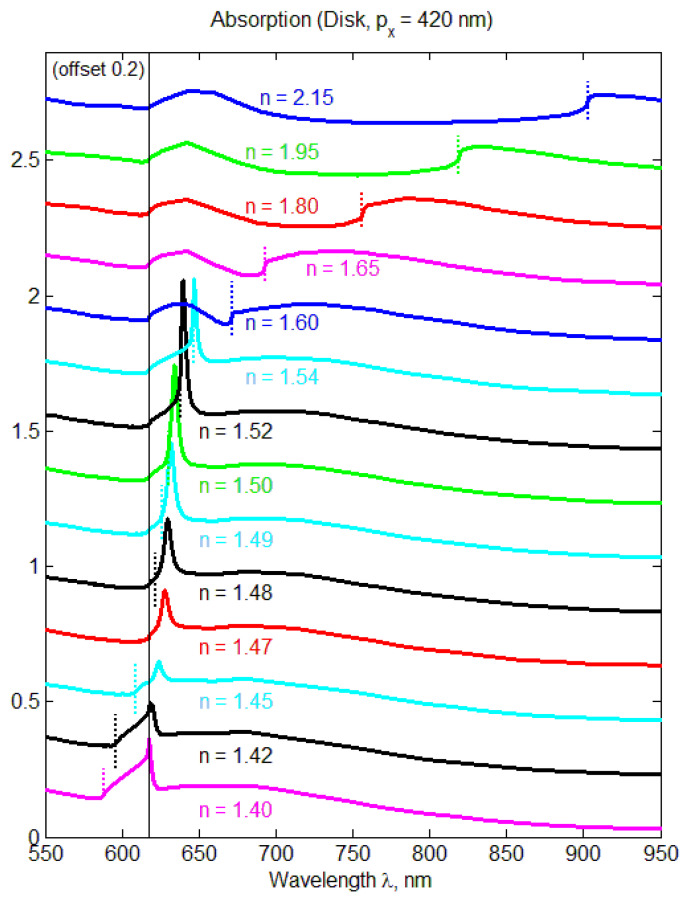
Absorption in a gold nanoparticle array facilitated by the lattice resonance for various refractive indices of the substrate *n*. Periods are $p_{x}=$ 420 nm and $p_{y}=$ 250 nm, a disk with the radius $R_{d}=$ 85 nm and height H= 50 nm, and the refractive index of the superstrate is $n_{s}=$ 1.47. The dotted color lines denote a wavelength of Rayleigh anomaly, that is, where the resonances in the substrate are expected, *i.e.,*$\lambda_{\mathrm{RA}}=np_{x}$, and the solid black line corresponds to the resonance in the superstrate, *i.e.,* $\lambda_{\mathrm{RAs}}=n_{s}p_{x}$. Light incidence is normal to the substrate, and electric field *E* is along the *x*-axis. For clarity, each absorption spectrum is vertically shifted by 0.2. Reproduced with permission from [69]. Copyright 2018 American Chemical Society.

Lattice resonances arise from the coherent interaction between plasmonic nanoparticles in a periodic array and can significantly enhance the local electric field. Plasmonic nanoparticle lattices offer significant field enhancement and nanoscale confinement, allowing for stronger light-matter interaction, increased spontaneous emission rate, and higher nonlinearity and absorption (Figure 4). Thus, such periodic metastructures, including metamaterials and metasurfaces, are smartly designed and engineered materials that have properties unattainable with natural materials. Additionally, plasmonic metasurfaces, which are two-dimensional nanoantenna arrays, provide subwavelength field localization.

Due to the confinement of light on the nanoscale, metasurfaces offer exceptional light manipulation and unique spectral features, often coinciding with resonances of high quality factor [60,61,62,63]. The behavior of lattice resonances in periodic structures is strongly influenced by the angle and polarization of the incident light under oblique incidence, which results in notable alterations in the spectral response and distribution of the electromagnetic fields [64]. The spectral behavior of plasmonic nanoparticles within the periodic array’s unit cell can also be analyzed numerically using various methods, such as finite-difference time-domain simulations or rigorous coupled wave analysis.

Recently, nanostructures that display strong coupling processes, namely, Fano resonances and Rabi splitting, have generated a considerable amount of growing interest. Rabi splitting is a unique form of strong coupling phenomenon that surpasses the dissipation rates of the system, unlike regular mode couplings. As a result, energy is exchanged coherently between the atom and the cavity. Researchers have conducted extensive investigations about Rabi splitting in various quantum and semiclassical systems, such as quantum-dot microcavity and emitter-plasmon systems, due to its potential promise and many applications in quantum information processing. Strong coupling due to Rabi splitting has been observed in plasmonic nanostructures and electromagnetic metamaterials.

The multipole decomposition applications for investigating directional light scattering by single nanoparticles and structures in a finite spatial region have been developed [65,66]. Even for large scatterers, the long-wavelength approximation’s multipole decomposition provides better convergence than exact multipole decomposition based on spherical harmonics expansion. To examine the contribution of the multipole moments in resonant excitations, radiation, and scattering of electromagnetic waves, multipole decomposition is widely used [67,68]. Periodic nanoparticle arrays have attracted more attention in recent years due to the narrow spectral resonances and tunable resonance positions that can be achieved through the lattice effect. The effective polarizabilities of multipoles and the scattering spectra of the structures have been examined, and analytical models are obtained for the coupled dipoles and quadrupoles.

Theoretical investigations have been carried out on the collective resonances that occur in plasmonic nanoparticle arrays, where the electric dipole moment is aligned along the lattice wave propagation. The resonant features of the arrays have been analyzed by examining the electric quadrupole and magnetic dipole moments of gold nanosphere using both semianalytical calculations of coupled multipole equations and rigorous numerical simulations [69,70,71,72]. It is instrumental to examine the impact of particle size and shape (spheres and disks) on electric and magnetic multipoles in both homogeneous and nonhomogeneous environments [69]. Results indicate that the nonresonant electric quadrupole and magnetic dipole moments of a single particle are significantly enhanced in the periodic lattice at the wavelength of collective resonance excitation. Additionally, enhanced electric quadrupole and magnetic dipole moments lead to reflection comparable to the electric dipole, satisfying the generalized Kerker condition, and resulting in strong magnetoelectric coupling at the lattice-resonance wavelength.

The focus of the analysis is often on the bright and dark modes excited in the nanoparticle array, with the mode interplay and coupling resulting in asymmetric spectral profiles, also known as Fano resonances [73,74,75]. Strong coupling between plasmonic states leads to energy level splitting, which is observed as Rabi splitting due to mode coupling facilitated by the lattice. Excitation of lattice resonances can enhance the Kerker effect, making it possible to observe and control the scattering properties of nanoparticles [76].

In Ref. [77], a comparison is made between analytical and numerical multipole decomposition outcomes near lattice resonances in two-dimensional periodic arrays of plasmonic and dielectric nanospheres. The results show that the exact multipole decomposition agrees well with simulations and requires only a small number of multipoles for accurate representation. These findings provide important insights into the validity and accuracy of multipole decomposition for analyzing the optical properties of periodic nanoparticle arrays, particularly around the lattice resonance.

### 5.1. Photoelectron Emission

**Figure 5:**
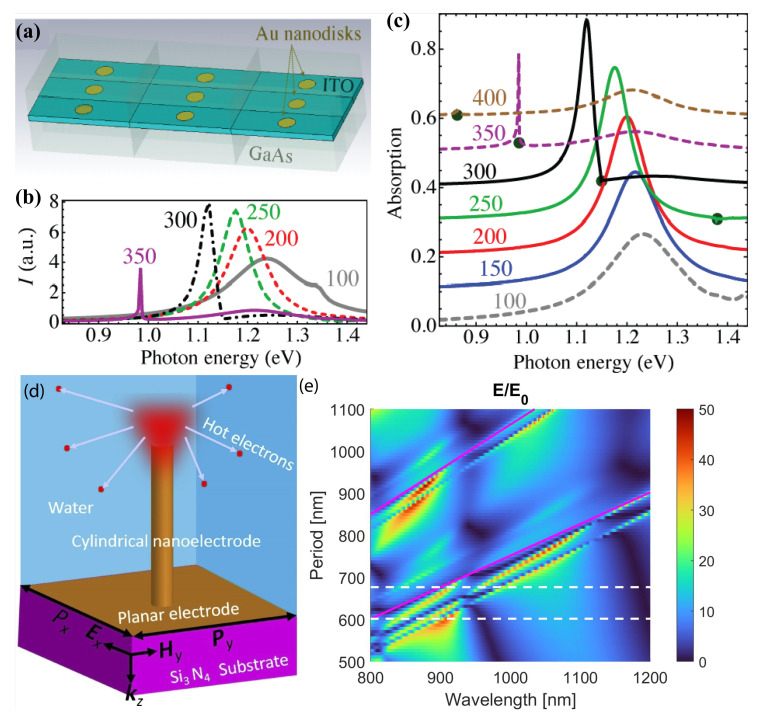
(**a**) Schematics, (**b**) photocurrent spectra, and (**c**) absorption spectra for the nanostructure designs with various array periods $p_{x}$. The nanodisk array is embedded in a transparent conductive oxide film and sandwiched between galium arsenide layers, providing a gold - galium arsenide interface at the front and back facets of the nanodisk. Incident light is *y*-polarized. The nanodisk radius is r= 25 nm and the height is h= 18 nm. Period $p_{y}$ along the *y*-direction is fixed to 100 nm. In panel (**b**), for clarity, each absorption spectrum is vertically shifted by 0.1 when the array period changes by 50 nm. (**d**,**e**) Nanostructure design aimed at improving hot-electron generation in nanoelectrodes. (**d**) Unit cell. The upper half-plane of the nanotube and planar electrodes are situated in water and have been positioned on a silicon nitride substrate. (**e**) The enhancement of electric field $\left(E/E_{0}\right)$ in an array of gold nanoelectrodes at a specific probe position varied for different lattice periods *P*. The enhancement can achieve values of up to 50 times, particularly in resonances that follow Rayleigh anomalies. The periods are $P=P_{x}=P_{y}$ in the *x*- and *y*-directions. The cylindrical gold nanoelectrode (nanotube) has a height of h= 1800 nm and an internal radius of 60 nm. The nanotube consists of walls that are 30 nm thick and is connected to a planar gold electrode of the same thickness. The wave propagates in the *z*-direction and is polarized in the *x*-direction. The solid magenta lines correspond to the Rayleigh anomalies (1,0) and (1,1). (**a**–**c**) Reproduced with permission from [78]. Copyright 2014 Springer Nature. (**d**,**e**) Reproduced from [80].

Plasmonic nanoparticle arrays exhibit collective lattice resonances that can be leveraged to amplify photoelectron emission in photodetectors and solar cells. The interdependence between narrow-band lattice resonances and wider-band individual-particle excitations is of paramount importance. It leads to stronger local field enhancement and, therefore, increased photocurrent (Figure 5a–c). These nanostructures can be used to design new photodetectors with a tunable spectral response and solar cells with increased efficiency [78,79].

Plasmonic hot electrons can be generated more efficiently in metal nanostructures by exciting nanoparticle plasmonic resonances. One can take the next step and arrange these nanoparticles into a periodic array and thus facilitate the enhancement of electric fields. The recent study [80] demonstrates that a nanostructure can be designed to enhance plasmonic hot electron generation from gold nanoelectrodes arranged periodically due to collective lattice resonances near Rayleigh anomalies (Figure 5d,e). This design can guide the development of plasmonic nanostructures with nanoelectrodes for efficient hot electron injection in an aqueous environment.

### 5.2. Nonlinear Response

**Figure 6:**
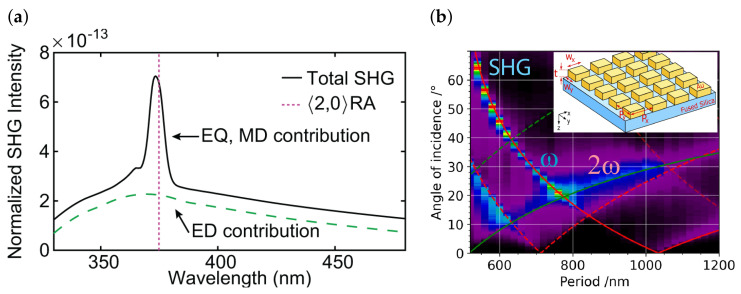
(**a**) Relative strength of the second harmonic generation in the nanoparticle lattice. The second harmonic generation spectrum shows a narrow resonance peak at 375 nm with an *x*-polarized incident electric field, corresponding to electric-quadrupole- and magnetic-dipole-multipole resonance at 750 nm. The (2,0)-order Rayleigh anomaly wavelength is denoted by the vertical dotted line, and the dashed line represents the electric dipole contribution at the fundamental wavelength to the second harmonic generation. (**b**) Measured power of the second harmonic generation stemming from the transmission into the 0th diffraction order. It is normalized with respect to the plot’s absolute maximum. The overlaid lines demonstrate the Rayleigh anomaly for the diffraction order $\left|m\right|=$ 1, with solid lines denoting conditions in air and dashed lines in fused silica. The corresponding wavelengths are represented by red (pump $\lambda_{\omega }=$ 1032 nm) and green (second harmonic $\lambda_{2\omega }=$ 516 nm) lines. The Rayleigh anomaly with $\left|m\right|=$ 2 is responsible for the partially visible branches at larger periods. The range of double resonant conditions with simultaneous excitation of both the fundamental and second harmonic surface lattice resonances have been examined. This has involved the creation of several two-dimensional rectangular arrays of centrosymmetric gold nanobars on fused silica, with varying lattice periods in one direction while keeping the localized surface plasmon resonance constant. The second harmonic generation has been measured at different angles of incidence and linear polarization directions from these metasurfaces. (**a**) Reproduced from [82]. (**b**) Reproduced with permission from [84]. Copyright 2022 by Optica Publishing Group.

Lattice resonances, which arise from the periodic arrangement of nanoparticles in the array, can strongly couple with the plasmonic resonances of the individual particles, resulting in further enhancement of nonlinear effects. Thus, plasmonic nanoparticle arrays exhibit strong nonlinear optical responses due to the collective excitation of multiple plasmonic resonances (Figure 6a). The excitation of multipole resonances, such as dipole, quadrupole, and octupole modes, leads to enhanced nonlinear effects in these arrays [81,82,83]. By controlling the size, shape, and spacing of nanoparticles, it is possible to tailor the lattice resonances and achieve efficient excitation of higher-order multipole modes, such as quadrupoles and octupoles. These higher-order modes can lead to enhanced nonlinear optical effects, such as second-harmonic generation and sum-frequency generation, making plasmonic nanoparticle arrays with lattice resonances a promising platform for nonlinear optics applications.

Recently, a parametric study has been conducted to investigate the impact of surface lattice resonance on second harmonic generation [84]. The left–right symmetry breaking associated with a tilted incident wavefront has been utilized to facilitate the necessary noncentrosymmetry required for second harmonic generation (Figure 6b). The role of double surface lattice resonance in maximizing SHG in plasmonic metasurfaces has been experimentally demonstrated. The parametric study on centrosymmetric two-dimensional nanobar arrays has confirmed that local maxima of SHG occur in correspondence to the surface lattice resonance either at ω or 2ω, leading to a further enhancement of frequency conversion when both pump and second harmonic surface lattice resonance are excited simultaneously. The double resonance was created by tilting the metasurface with a specific period in just one direction. The close connection between the linear and nonlinear response of the metasurface has been theoretically confirmed using the nonlinear inverse scattering approach.

### 5.3. Lossy Materials

**Figure 7:**
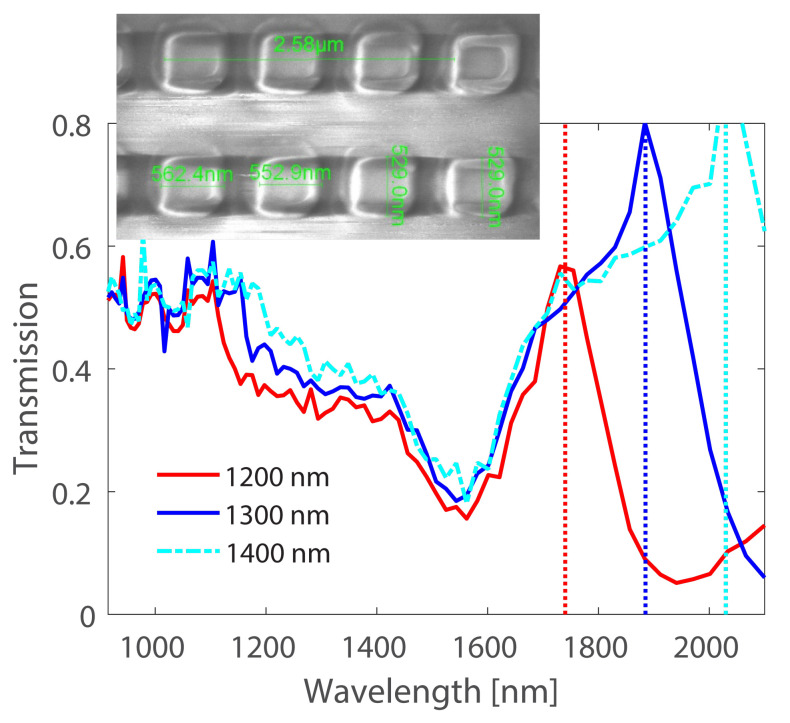
Experimental measurements for the transmission through nanoparticle array for $d_{x}$ = 1200, 1300, and 1400 nm. The vertical dotted lines denote the spectral position of the Rayleigh anomaly. Inset: SEM image of the array. The nanoparticle array is considered in the homogeneous (uniform) surrounding medium with a refractive index *n* = 1.45 (corresponds to glass). The cuboid nanoparticles are made of nickel with the dimensions $a_{x}=a_{y}$ = 530 nm and $a_{z}$ = 300 nm. The incident plane wave is linearly polarized: it is normal to the array plane, propagates along the *z*-direction, and has an electric field along the *y*-direction. The two-dimensional infinite arrays have periods $d_{x}$ and $d_{y}$ = 1000 nm in the *x*- and *y*-directions, respectively. Reproduced from [86].

A novel method for achieving strong resonances in nanoparticle arrays with high optical losses has been proposed in a recent study [85], which holds the potential for efficient light manipulation in ultra-thin optical elements, sensing, and photovoltaic applications. Strongly localized nanoparticle resonances can be excited in materials with a large imaginary part of permittivity [86,87]. Arranging such lossy particles in a periodic array allows for stronger collective array resonances that can be easily tuned in a broad spectral range, mainly by the array period (Figure 7). Transition metal dichalcogenides, including tungsten disulfide WS${}_{2}$, possess large permittivity and support well-defined Mie resonances [88,89]. A periodic array of WS${}_{2}$ nanoantennas can control different multipole resonances via the lattice period [85], making it a potential tool for future nanophotonic devices.

### 5.4. Complex Nanoantennas

Three-dimensional optical nanostructures (metamaterials) are often designed by stacking together thin layers of metal–dielectric materials to create a composite material possessing exceptional functionalities to control, confine, and enhance light-matter interactions at the subwavelength scale. Despite these exceptional abilities, the composition of three-dimensional nanostructures (metamaterials) leads to a significant amount of losses and creates a lot of fabrication challenges. These drawbacks make three-dimensional plasmonic nanostructures (metamaterials) unsuitable for integration into photonic device applications. Besides their light weights, two-dimensional metasurfaces (nanostructures) have been designed to have the same exceptional functionalities and to overcome the fabrication challenges possessed by three-dimensional metamaterials.

An example of a plasmonic metasurface is the multisegment silver-silicon nanopillar, which exhibits multiple mode excitations that are similar to those found in complex interfaces of multilayer metal and dielectric structures [90]. The numerical study investigated the spectral response of single and paired nanopillars within the unit cell of the periodic array. The analysis was carried out using computational methods, which allow for a thorough examination of the optical behavior of the plasmonic metasurface. Such nanostructures provide insight into the plasmonic modes that are excited within the nanopillar array, as well as their interplay and coupling, which leads to asymmetric spectral profiles, often referred to as Fano resonances.

The enhancements in the electromagnetic fields give rise to the formation of bright modes (field enhancement), whereas the suppression of the electromagnetic field produces dark modes (field suppression). The effective coupling of the incident light and the bright modes results in the enhancement of the magnitude of the absorption. The interaction of the bright mode and the dark mode gives rise to an asymmetric resonant profile in the absorption spectra resulting in Fano resonance. The results also demonstrate the ability to realize three optical processes, namely Fano resonances, Rabi splitting, and bound state in the continuum (BICs), due to the hybrid silver–silicon multilayer design of the metasurface.

## 6. Photovoltaics

The idea of using plasmonic effects in metallic nanostructures to augment the photovoltaic properties in photodetectors has been recently proposed after the rapid development of nanoscale fabrication and characterization. Owing to plasmonic phenomena, which originate from the interaction between light waves and the motion of free electrons in metals, plasmonic nanostructures offer unprecedented versatility in guiding, confining, and manipulating light at the nanoscale. Therefore, there is strong promise in using plasmons for applications related to advanced light detection and solar energy harvesting.

For example, plasmonic nanoparticles on the surface of a semiconductor layer provide strong antireflective properties in the visible spectral range and, thus, improve the efficiency of photovoltaic devices and solar cells. It has been shown that silver nanoparticles enable up to 30% increase in the overall absorbance within the semiconductor layer [91]. The directional scattering and Kerker effect can be utilized in designing antireflective coatings for solar cells, as it enables selective scattering of light in a way that reduces reflection and enhances absorption. Thus, plasmonic nanostructures can function as coatings that minimize unwanted reflection from solar cells, as light-trapping structures that spatially redistribute light to optimize its harvesting, or as resonant arrays that channel more light into localized plasmonic modes to enhance photocurrent generation.

Following a period of intense studies, plasmonic photovoltaics is now a rapidly maturing scientific discipline with the primary target of improving efficiency and decreasing the cost of solar cells, as well as developing new concepts in photovoltaic devices. In all these studies, the role of free electrons in metal in conventional plasmonics has been limited to their interior motion inside each metallic inclusion, as a part of the electron gas spatially co-located with metallic nanoparticles. Several recent works have considered such enhanced photoemission of electrons in order to facilitate narrow-band long-wavelength photodetection. It is worth stressing that even though this assumption was found to be applicable to many practically relevant scenarios, and even though conventional plasmonics has already offered to enable discoveries such as ultrahigh local field enhancement and deeply subwavelength light confinement, it has recently become clear that the interaction of light with plasmonic nanostructures offers a much richer physics than what can be described by a purely electrodynamic paradigm with the Drude model. On the few-nanometer scales, the hydrodynamic model of the internal electron ensemble behavior (nonlocal response) has proved essential in describing plasmonic properties.

Another extension of free electron dynamics is relaxing the “inside-particle” constraints on the motion of electrons and accounting for the process of electron photoemission from metallic nanoparticles into the surrounding medium under the driving action of the electromagnetic fields. Such processes themselves have known since the invention of the vacuum tube, must inevitably be present whenever strong local fields are involved, for example, those typically generated in resonant plasmonic structures [80]. Efficient plasmonic hot electron generation in metal nanostructures can be achieved by exciting nanoparticle plasmonic resonances. In the scenario when nanoparticles have a common interface with semiconductors, these intense local fields facilitate the enhanced photoelectron emission through the Schottky barrier at the metal/semiconductor interface. This allows long-wavelength photons with energy below the bandgap insufficient for direct band-to-band absorption in semiconductors to contribute to the overall photocurrent, possibly improving the performance of photovoltaic elements [92,93].

Current state-of-the-art results with photoconductive nanostructures appear promising. They also show that there are strong demands and many possibilities for further research in this field. First, there is much promise in extending plasmon-assisted photoemission studies from simple-shaped nanoparticles to more complex-shaped nanoparticles and nanoparticle clusters, whose plasmonic properties are known to harbor rich physics that can be straightforwardly translated into the photoconductive domain. For instance, recent results on plasmonic nanodisk dimers indicate the further enhancement of local fields in the resonant plasmonic modes compared to a lattice of single particles. The resonant frequency tuning in such arrays can also be performed by manipulating the dimer sizes and arrangements. Second, there is a demand for a systematic photoconductivity approach with a solid theoretical foundation to mature the concept of photoconductive nanostructures. Followed by the development of a robust numerical platform that combines two solvers for an electromagnetic problem and electron photoemission processes, the concept will become a practically viable technological tool.

Aside from the indirect action of improving light harvesting by spatial redistribution, the plasmonics effects can directly contribute to photoelectric effects. Internal individual resonances, such as localized plasmonic resonances of metal nanostructures, enhance light absorption by orders of magnitude and, eventually, can cause the emission of “hot” photoelectrons from nanostructures [92,94,95,96,97]. This effect—plasmon-assisted photoconductivity—has recently emerged as a promising research direction [98,99,100,101,102] and can potentially bring about a variety of photosensing, photodetection, photochemical, and photovoltaic applications [101,102]. Furthermore, one can combine the existing light-trapping capabilities of plasmonic nanoparticles with the photoelectric effects related to the external generation of “hot” electrons. This approach has the potential for a broader range of applications in light-harvesting devices, such as photoconductive plasmonic photodetectors, solar cells, photochemical cells, and other related technologies [78,98,103,104,105,106,107].

Promising approaches involve merging two concepts—optical nanostructures and plasmonics-enhanced photoconductivity—into one advanced design of photoconductive nanostructures, which provides a very fruitful platform for next-generation plasmonic devices such as photovoltaic cells and photodetectors. At the same time, photoconductive nanostructures bring about a fundamentally new way of manipulating light and tailoring light-matter interaction at the nanoscale. As an illustrative example, embedding an array of nonsymmetric uniformly oriented plasmonic nanoparticles in a semiconductor matrix results in the appearance of a net flux of photoemitted electrons, and in turn, of a photoelectromotive force under homogeneous external light illumination. Thus, a carefully prepared metal-semiconductor composite exhibits a pronounced bulk photoelectric effect with the possibility to be scaled across the optical frequency range by varying the meta-atom composition.

In a broader context, the very concept of such photoconductive nanostructures manifests a new direction in applications, where fundamentally distinct physical properties (classical optical and quantum mechanical) are made to work together to facilitate efficient photodetection, photovoltaics, and photochemistry. The concept of photoconductive nanostructures has a far-reaching perspective of designing and fabricating unique materials where the abilities to change conductivity, generate a photo-emf, and produce a photocurrent in a closed circuit under illumination will be combined with already renowned properties of nanostructures such as polarization shaping, superresolution, and anomalous refraction. There have also been suggestions to utilize photoelectron emission from graphene-based plasmonic structures [108], where a very low work function $W_{b}$ ∼ 0.2 eV [109] can give rise to the significantly increased photocurrent. Taken together, the concept can be invoked in various focus areas in technology and industry.

## 7. Light Sources

**Figure 8:**
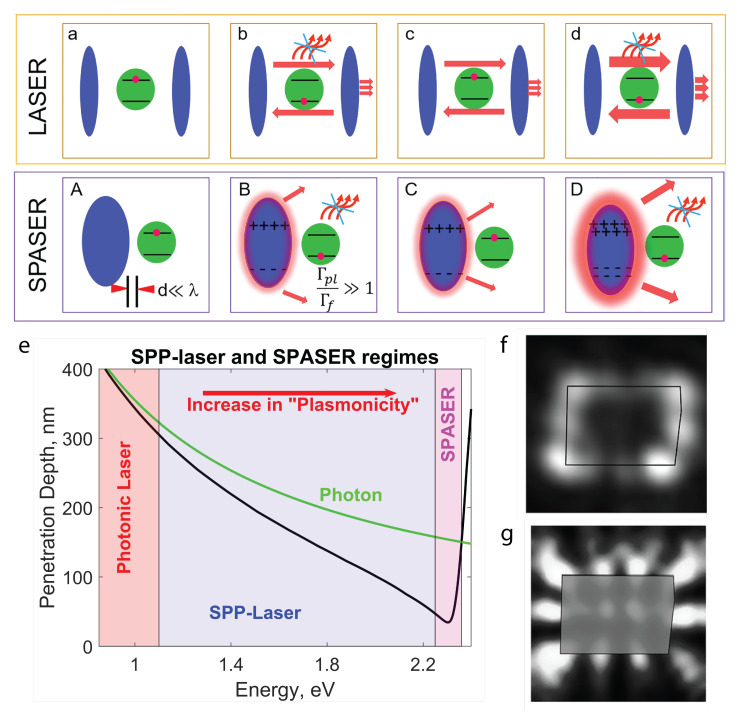
(**a**–**d**) Key differences and similarities of the conventional laser (top row) and the SPASER (bottom row). The green circle shows an emitter, the top panels show blue mirrors, and the bottom panels show plasmonic metal. (**A**) and (**a**) Excited emitter in the cavity or next to the plasmonic nanostructure at a distance *d*, much smaller than wavelength λ. (**B**) and (**b**) Spontaneous emission into cavity mode or energy transfer to plasmonic mode, dominating over emission into free space. $\Gamma_{pl}$ and $\Gamma_{f}$ are the rate of energy transfer to plasmons and emission into free space, respectively. (**C**) and (**c**) Emitter re-excited in the presence of the cavity or plasmonic mode. (**D**) and (**d**) The existing cavity mode or plasmonic oscillation triggers the excited emitter to emit the same photon (**d**) or plasmon (**D**), rather than emitting spontaneously into the surrounding free space. The repetition of this step leads to lasing or spasing. For SPASER (B through D), far-field emission from the plasmons is also possible. This far-field radiation originates from the plasmon oscillation in the metal nanostructure, rather than from the emitter directly. (**e**) The of plasmonic field penetration versus energy for a metal–dielectric interface. In the calculations, the metal is silver, described by experimental data [118], and the dielectric has permittivity $\varepsilon_{c}=12$. (**f**,**g**) Maps of the plasmonic laser when the pump power exceeds the threshold, ∼3.6 $P_{\mathrm{th}}$, where $P_{\mathrm{th}}$ = 3.5 mW is the peak pump power at the lasing threshold. (**f**) Stimulated emission directing more light to the single lasing mode. The single-mode operation in a plasmonic nanolaser, resulting from the stimulated emission directing more light to the lasing mode, leads to a more pronounced distribution of the lasing mode compared to other cavity modes. (**g**) Near-field distribution of the absolute value of an electric field |E| of the lasing mode obtained with 3D full-wave numerical modeling. (**a**,**d**) Reproduced from [116]. (**f**,**g**) Reproduced with permission from [117]. Copyright 2018 American Chemical Society.

The use of metallic or plasmonic cavities has brought about a significant change in the development of semiconductor lasers, leading to a remarkable reduction in size. Surface plasmons can be directly generated on a plasmonic (metal) nanostructure and amplified by a dielectric with gain. Importantly, in this case, a feedback mechanism in the nanostructure allows for surface plasmons to acquire coherence [110]. The amplified optical field is confined in the metal–dielectric interface and typically cannot be outcoupled without specially designed optics. Such plasmonic light sources utilize field confinement to produce intense optical energy on a subwavelength scale. The energy can also be changed on a very short time scale [111,112,113,114]. These light sources rely on surface plasmons the same way as lasers rely on photons, with one dramatic difference. SPASER stands for surface plasmon amplification by stimulated emission of radiation. It is a nanoscale device that combines the properties of plasmons and lasers (devices that amplify light through stimulated emission) to create an ultra-compact and efficient light source. As the name suggests, SPASERs operate by exciting plasmons in a plasmonic nanostructure, which can then be amplified by stimulated emission to produce coherent light. A detailed comparison of the conventional laser and the SPASER is shown in Figure 8, panels a through d. Photons are electromagnetic oscillations that cannot be localized in space to a size less than a quarter wavelength. In sharp contrast, the surface plasmons are quanta of electromechanical oscillations. Consequently, the source is nanoscopic and scalable on a characteristic size of ∼2–50 nm.

Nanolaser design employs metallic structures to confine optical modes on the nanoscale, as illustrated in Figure 8e. Here, the energy range is approximately divided into three regions: photonic laser (or conventional laser), SPP-Laser, and SPASER. These regions are identified depending on the “plasmonicity”, which is the degree of proximity to the surface plasmon resonance. The latter manifests as a drastic drop in the penetration depth (≈2.25–2.35 eV). The line “Photon” shows the penetration depth taken at 0.8 eV and multiplied with the coefficient (0.8 eV)/(excitation energy in eV) to adjust it for the change in the energy of plasmon excitation. In the “Photonic Laser” regime, the metal functions as a perfect reflector because of the negative and large absolute value of the real part of the metal permittivity. Thus, wave energy is expanded outside the metal. In the other case, surface plasmons are excited, and photons do not play any significant role. Moving towards the surface plasmon resonance, plasmonic effects or “plasmonicity” increases, as indicated by the arrow [115,116].

In order for a plasmonic nanolaser to exhibit lasing behavior, its emission pattern must correspond to an eigenmode of the nanolaser resonator. Thus, the emission pattern serves as a crucial means for confirming lasing behavior and identifying the specific lasing eigenmode [117]. Above the threshold, the stimulated emission directs more light to the lasing mode, resulting in a single-mode regime and a more pronounced distribution of the lasing mode compared to other resonant modes (Figure 8f). Once the lasing regime is reached, the emission distribution remains unchanged despite variations in pump power. The simulated near-field pattern of the absolute value of the electric field |E| in the lasing mode, as shown in Figure 8g, exhibits the same main features as the experimental emission pattern. This simulation aids in optimizing the performance of plasmonic nanolasers by providing insight into the distribution of the electric field in the lasing mode.

Plasmonic processes can play an important role in the operation of lasers and light sources by enhancing light-matter interactions, improving energy transfer efficiency, and increasing light extraction. Here are a few examples of plasmonic processes that are relevant to lasers and light sources:

- Plasmon-enhanced absorption (PEA): Plasmonic nanostructures can enhance the absorption of the incident light, leading to increased energy transfer efficiency and improved laser performance. For example, similar to how plasmonic nanoparticles can be used to increase the absorption of light in thin-film solar cells, they can enhance the absorption of laser light and strengthen feedback in solid-state laser materials.
- Plasmon-induced resonance energy transfer (PIRET): PIRET is a process in which energy is transferred from a plasmonic nanostructure to a nearby molecule or chromophore, leading to enhanced emission or other photophysical processes. This process can be used to enhance the efficiency of light-emitting diodes (LEDs), or to improve the performance of fluorescence-based sensors.
- Surface plasmon resonance (SPR): SPR is a phenomenon in which light interacts with the collective oscillations of electrons in a thin metal film, leading to strong absorption or scattering of light at certain wavelengths. This process can be used to create plasmonic waveguides or resonators, or to improve the sensitivity and selectivity of optical biosensors.
- Plasmonics-assisted light extraction: Plasmonic nanostructures can also be used to enhance the extraction of light from LEDs or other light sources, by increasing the amount of light that is coupled out of the device and into the surrounding environment. This process can improve the efficiency and brightness of LEDs, or enable the use of low-cost, flexible, and lightweight organic-LED displays.

Overall, plasmonic processes offer a range of opportunities for enhancing the performance and functionality of lasers and light sources, and are an active area of research in the field of photonics.

The Purcell effect is a phenomenon in which the spontaneous emission rate of an excited emitter (such as an atom or quantum dot) is enhanced by its coupling to a resonant optical cavity, resulting in a shorter emission lifetime and increased photon emission efficiency. The process behind the Purcell effect is the modification of the local density of optical states, which affects the rate of emission and reabsorption of photons by the emitter. The Purcell effect can enhance the spontaneous emission rate of plasmonic nanolasers by coupling the emitter to a resonant optical cavity, resulting in improved efficiency and reduced lasing threshold.

Most of the experimental demonstrations of plasmonic sources practically belong to the three basic types: (i) nanoparticle of metal surrounded by a nanoshell of gain medium [119,120]; (ii) nanoparticle of the gain medium on an extended plasmonic metal [12,121,122,123,124,125,126]; and (iii) a plasmonic metal nanoparticle on an extended gain medium and electrical pump. It provides a source of coherent and intense local optical fields. Designs that include a tip focusing provide several advantages. The tip monitors the local electromagnetic fields of the source and can also (adiabatically) focus the pump radiation into the gain medium [127,128]. The design offers a convenient scheme to explore a variety of excitonic nanomaterials and position-dependent diagnostics of the nanomaterials, including the edges, by simply moving the tip from one sample to another. Such experimental arrangement allows directly measuring the highly confined plasmonic modes at the tip-sample junction via tip backscattering. Plasmonic light sources exhibit nonsaturating linear light-pump characteristics, and they offer means to compensate for loss as the coherently amplified light is self-sustained.

Another type of plasmonic nanoscale laser relies on the collective resonances in nanoparticle arrays to generate coherent and intense light (discussed above in Section 5). The development of plasmonic nanolasers has attracted significant attention due to their potential for a wide range of applications, including sensing, data storage, and nanophotonics. In these nanolasers, the collective resonances of nanoparticles in a periodic array create a strong localized electromagnetic field that amplifies the light emission from the active material (usually a gain medium such as dye or quantum dots) located in the proximity to the nanoparticles and the gaps between them [129,130]. The resonant coupling between the nanoparticles leads to the formation of SPPs, which can be confined to subwavelength regions and provide enhanced light-matter interactions.

To achieve lasing in plasmonic nanoparticle arrays, several conditions must be met, including a sufficiently high quality factor of the resonant modes, a strong enough feedback mechanism to sustain the lasing action, and an efficient coupling of the gain medium to the resonant modes. Recent studies have demonstrated the successful fabrication of plasmonic nanolasers based on various types of nanoparticle arrays, including metallic nanodisks, nanorods, and nanocubes. These nanolasers exhibit a range of characteristics, including ultra-low threshold, high efficiency, and tunability of the emission wavelength. Plasmonic nanolasers based on collective resonances in nanoparticle arrays represent a promising platform for realizing ultra-small, low-power, and tunable light sources with potential applications in diverse areas of nanophotonics and optoelectronics.

## 8. Sensors

**Figure 9:**
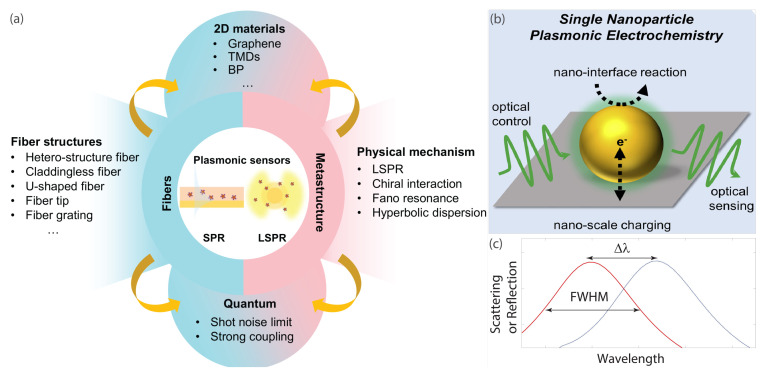
The development of surface plasmon sensors has made significant progress in recent years, particularly in planar metastructures and optical-fiber waveguides. (**a**) Broad application of plasmonic nanostructures in sensing enabled by their remarkable ability to confine light at the nanoscale and support the excitations of surface plasmon resonances (denoted ‘SPR’) or localized surface plasmon resonances (denoted ‘LSPR’) with associated effects and sensitivity to the environment. Optical nanostructures with LSPR, hyperbolic dispersion, Fano resonance, and integration with two-dimensional (denoted ‘2D’) materials in the metastructure platform have recently been introduced. The integration of optical-fiber sensors with LSPR/SPR structures and two-dimensional materials has been achieved. Quantum plasmonic sensing has surpassed the conventional shot-noise limit, demonstrating significant progress in the field. Although these surface plasmon designs bring countless opportunities in this field, they also come with challenges, which are discussed in the main text. (**b**) A simple plasmonic sensing geometry consists of a nanoparticle on a substrate. (**c**) When illuminated with light of a particular wavelength, the nanoparticle exhibits a characteristic scattering spectrum that follows a Lorentzian lineshape with a clearly defined resonance wavelength $\lambda_{r}$ and full width at half maximum (FWHM). (**a**) Reproduced from [5]. (**b**) Reproduced with permission from [132] Copyright 2018 by Elsevier.

The main components of sensors include target, recognition, and transducing elements. Conventional sensing methods include electrochemical, chromatography, or mass-sensitive types and span various possible applications. In turn, surface plasmon sensors have become an important tool for various applications, including biology, chemistry, and environmental monitoring, due to their extraordinary sensitivity based on SPR or LSPR effects. Plasmonic sensing has numerous applications in areas such as biomedical sensing, environmental monitoring, and food safety. It offers high sensitivity, label-free detection, and real-time monitoring capabilities, making it a promising tool for a wide range of analytical applications. Over the past few years, remarkable advancements have been made in surface plasmon sensors, with special emphasis on planar metastructures and optical-fiber waveguide configurations (Figure 9).

Plasmonic sensing is a powerful technique for detecting and analyzing biomolecules, gases, and other analytes at extremely low concentrations [5,131,132,133,134,135]. The technique relies on the interaction of light with metallic nanostructures, typically gold or silver nanoparticles, that exhibit LSPRs. These resonances arise from the collective oscillation of free electrons in the metal when excited by an electromagnetic field, resulting in strong absorption, scattering, reflection, and/or transmission of light at specific wavelengths.

The position of the resonance peak depends on the size and shape of the nanoparticle, as well as the refractive index of the surrounding medium [136,137,138]. Plasmonic sensors operate by monitoring changes in the resonance wavelength $\lambda_{r}$ that occur when analyte molecules bind to the surface of the nanoparticle, altering the local refractive index. The amount of wavelength shift $\Delta \lambda =\lambda_{r}^{*}-\lambda_{r}$ is proportional to the change in refractive index Δn (see Figure 9c), and the system’s sensitivity is typically given by S=Δλ/Δn. To account for differences in FWHM between different nanoparticle geometries and experimental setups, FoM is often used to measure sensor performance. The value of FOM is defined as the sensitivity normalized by the FWHM, or FoM = *S*/FWHM.

In plasmonic sensors, various phenomena, such as BIC, Fano resonance, and the Kerker effect, can be utilized to enhance detection and sensing capabilities [139]. Bound state in the continuum allows for the confinement of light in a subwavelength structure with almost perfect reflection, whereas Fano resonance enables high sensitivity and selectivity in sensing applications by utilizing the interference between a broad and a narrow resonance [140]. On the other hand, the Kerker effect involves controlling the scattering direction of an incident light by designing a resonant structure with specific geometries and materials. Together, these effects can contribute to the development of more advanced and sensitive plasmonic biosensors.

The metastructure platform has paved the way for various optical sensors utilizing LSPR, hyperbolic dispersion, Fano resonance, and integration with two-dimensional materials. Surface plasmon resonance sensors have been shown to exhibit high sensitivity due to the localized fields generated at the metal–dielectric interface. Hyperbolic dispersion sensors, which rely on the anisotropic permittivity of metal–dielectric multilayers, have demonstrated enhanced sensitivity and selectivity. Fano resonance sensors, which rely on the interference between a broad spectral feature and a narrow spectral feature, have been shown to have high FOM values. Integration of two-dimensional materials with plasmonic sensors has opened up new opportunities for sensing applications, as the electronic and optical properties of two-dimensional materials can be tuned by varying their thickness and composition.

In the optical-fiber platform, sensors integrated with LSPR/SPR structures and two-dimensional materials have been summarized. Surface-plasmon-resonance-based fiber sensors have demonstrated high sensitivity and selectivity, and have been used for various applications such as environmental monitoring and chemical sensing. Integration of two-dimensional materials with fiber sensors has also been shown to enhance their sensitivity and selectivity.

In addition to classical sensing approaches, recent advances in quantum plasmonic sensing beyond the shot noise limit have been introduced. Quantum plasmonic sensors rely on the quantum properties of light and can achieve higher sensitivity than classical sensors. These sensors can potentially enable new applications in quantum information processing and quantum sensing.

Despite the significant progress in the field of surface plasmonic sensors, there are still challenges and opportunities that need to be addressed. For example, improving the reproducibility and stability of plasmonic sensors, developing new sensing modalities based on plasmonic effects, and integrating plasmonic sensors with other technologies to enhance their performance. Addressing these challenges could lead to new sensing applications and enable advances in fields such as biomedical research and environmental monitoring.

### 8.1. Biosensors

Biosensor facilitates specific interaction, which results in recognition element capturing (detecting) analyte molecules. Subsequently, the recognition element experiences a change to its properties as a result of binding with the target molecule. Examples of the properties include conductivity, refractive index, current, resonance frequency, and polarization, and this change is converted to another signal with the help of a transducer. Five categories of viral biosensors can be identified [134]. These are immune, DNA, antigen, cell, and molecular imprinting, categorized based on the type of recognition element and virus target.

Surface-plasmon-resonance biosensors based on plasmonic nanostructures have emerged as reliable tools for the detection and analysis of biological and chemical molecules [141,142,143,144,145,146,147,148,149]. The chip-based sensors typically comprise a glass substrate coated with (∼50 nm) of metal and a sensing layer. Such design enables the recognition of analytes flowing through a microfluidic channel near the sensing layer.

In plasmonic biosensors, surface plasmons are excited by light to detect changes in the refractive index of the interface where the molecules are located. In this process, surface plasmons couple to the metallic surface to compensate for mismatched momentum. Various techniques are applied to add the required momentum, including prism or grating coupling, and other optical elements. Surface-plasmon-based biosensors have attracted considerable attention due to their high sensitivity, real-time detection capability, and label-free operation. These biosensors operate by detecting changes in the refractive index of the medium adjacent to the sensor surface, caused by the binding of analyte molecules to the functionalized sensing layer on the metal surface.

One of the main advantages of plasmonic biosensors is their label-free detection capability, eliminating the need for functionalization with multiple antibodies as required in conventional assays, such as enzyme-linked immunosorbent assays. Additionally, plasmonic biosensors allow for dynamic measurement of binding–unbinding kinetics, enabling the observation of reaction mechanisms occurring over the sensing surface. These sensors also offer high sensitivity for detecting low concentrations of analytes.

Despite their advantages, standard SPR chips have limitations, such as a requirement for transverse magnetic polarized light, low selectivity, and a small penetration depth of only ∼200–300 nm. Although the small penetration depth is advantageous for the sensing of small molecules or bioentities in the nanoscale vicinity of the plasmonic surface, it poses a challenge when detecting larger entities, such as bacteria or cells. In these cases, a large penetration depth of the plasmonic field is needed. To overcome this obstacle, modifications can be made to the chip design, such as using long-range SPR chips. These modified chips provide a higher penetration depth and improved selectivity by using a combination of surface plasmons and waveguide modes, which enhances the sensitivity and specificity of the biosensors.

The sensing layer can be tailored to specifically interact with the target biomolecules, such as proteins, DNA, or small molecules, through various immobilization methods, including physical adsorption, covalent binding, or affinity capture. When the target biomolecule binds to the sensing layer, it causes a change in the refractive index of the medium, which results in a shift in the resonance angle or wavelength of the SPR signal. The SPR signal can be measured using various detection schemes, such as angular interrogation, wavelength interrogation, or intensity measurement. These biosensors have been applied in various fields, including clinical diagnosis, environmental monitoring, food safety, and drug discovery. Plasmonic chiral nanostructures, e.g., metasurfaces [150], can be used as biosensors to detect the chirality of molecules, which is important for identifying biological molecules and monitoring chemical reactions.

In addition to conventional SPR biosensors, plasmonic biosensors based on other types of plasmons, such as localized surface plasmons, propagating surface plasmon polaritons, and guided-mode resonances, have also been developed. These biosensors offer different advantages and limitations, depending on the type of plasmon mode used and the sensing platform design. Overall, plasmonic biosensors based on surface plasmons have shown great potential for sensitive and specific detection of a wide range of biomolecules and analytes, and are expected to have significant impacts on various applications in the future.

**Biosensors for viral diagnostics.** Plasmonic biosensors are seen as a novel, highly sensitive approach to identifying viruses [135]. They allow for relatively simple procedures, minimal pretreatment of the target, and inexpensive equipment. When a plasmonic biosensor is used for detecting viruses, it detects the presence of specific viral particles or viral components in a sample. A plasmonic biosensor typically consists of a surface that is coated with specific molecules that can bind to the target virus or viral component of interest. When a sample containing the virus is added to the biosensor, the viral particles or viral components bind to the molecules on the sensor surface, causing a change in the local refractive index or electromagnetic properties of the surface. This change can then be measured and used to detect the presence of the virus. The specific viral component that is targeted by the biosensor can vary depending on the design of the biosensor and the virus being detected. For example, some biosensors may target specific viral proteins, whereas others may target viral RNA or DNA.

In the plasmonic biosensor for viral diagnostics, the detection is typically based on optical processes, such as the excitation of localized resonances, propagating surface waves, SERS, surface-enhanced fluorescence, and surface-enhanced infrared absorption spectroscopy. Plasmonic sensors have several advantages compared to conventional sensors, including real-time tracking of the binding dynamics, label-free detection, extensibility and maintainability, fast response, simple sample target treatments, and limited requirement for electrical components. More research is needed to increase the specificity of the binding surface by immobilizing the molecular-selective interface. Moreover, plasmonic sensors have issues with mass transportation and may suffer from misinterpreted detection for similar events.

### 8.2. Chemical Sensors

Chemical plasmonic sensors are a promising technology for detecting and quantifying the presence of chemicals and gases in various environments. These sensors work by monitoring changes in the optical properties of plasmonic materials when they come into contact with the target analyte. The sensing mechanism is based on the interaction between the analyte and the plasmonic nanostructure, which causes a shift in the plasmon resonance frequency and/or intensity. This shift can be detected using SPR and various optical techniques, such as spectroscopy or ellipsometry. Although there are some similarities between these two types of sensors, the design and operation of each are tailored to the specific application and environment in which they are used.

One key difference between chemical plasmonic sensors and plasmonic biosensors is the nature of the analyte [151,152,153,154]. In chemical plasmonic sensors, the analyte is typically a chemical compound or gas, whereas in plasmonic biosensors, the analyte is a biological molecule, organism, viral protein, RNA, or DNA. This difference has important implications for the design and operation of the sensor. For example, chemical plasmonic sensors often require the use of specialized coatings or functionalization layers to enhance the selectivity and sensitivity of the sensor. These coatings are designed to bind selectively to the target analyte, thereby enhancing the sensitivity and reducing interference from other molecules in the environment.

Another important difference between chemical plasmonic sensors and plasmonic biosensors is the operating environment. Chemical plasmonic sensors are typically operated in harsh or extreme environments, such as high temperatures, pressures, or chemical concentrations. In contrast, plasmonic biosensors are typically operated in aqueous or biological environments, which require different types of coatings and surface chemistries to ensure biocompatibility and prevent biofouling.

In addition to these differences, there are also important differences in the types of plasmonic materials and structures used in chemical plasmonic sensors compared to plasmonic biosensors. For example, chemical plasmonic sensors often use metal nanoparticles or nanorods, which exhibit strong plasmon resonances in the visible or near-infrared regions of the electromagnetic spectrum. These plasmonic materials are often combined with porous or mesoporous materials to increase the surface area and enhance the sensitivity of the sensor. In contrast, plasmonic biosensors often use planar or structured surfaces, such as gratings or nanoholes, which can be functionalized with biological molecules to selectively capture and detect specific analytes.

## 9. Discussion and Conclusions

In conclusion, the success of plasmonic applications relies on the underlying optical processes that occur in plasmonic nanostructures. These optical processes involve the interaction between light and the collective oscillation of electrons at the metal–dielectric interface, leading to a wide range of applications in various fields. The localized surface plasmon resonance is a fundamental process that can be exploited for various applications, such as sensing and spectroscopy, by taking advantage of the strong enhancement of local electromagnetic fields. Surface-enhanced Raman scattering is another important process that enables dramatic enhancement of the Raman signal, offering crucial analytical capabilities in chemistry, biosensing, and imaging.

Plasmon hybridization and Fano resonance are two additional optical processes that can be used in various applications, such as plasmon-enhanced light harvesting, plasmon-mediated energy transfer, and plasmon-induced hot electron generation. These processes arise from the coupling between plasmons in different nanostructures and the interference between a discrete resonance and a broad background continuum. Overall, manipulating the optical processes in plasmonic nanostructures provides a versatile platform for achieving various applications, including sensing, imaging, energy conversion, and catalysis. The continued development and optimization of these processes will enable new and exciting applications in the future.

Plasmonic nanoparticle arrays with lattice resonances have demonstrated promising applications in enhancing nonlinear optical phenomena due to their ability to excite multipole resonances. By tuning the size, shape, and spacing of the nanoparticles in the array, one can precisely control the lattice and plasmonic resonances, enabling tailored nonlinear optical responses for a wide range of applications.

Plasmonic nanostructures can help to increase conversion efficiency in light-driven devices through several mechanisms. Firstly, they can enhance light absorption by concentrating and trapping incident light within a small volume, increasing the amount of light available for conversion. Plasmonic nanostructures can also exhibit LSPR, resulting in a strong, resonant interaction between the nanostructure and incident light. This can lead to enhanced absorption, scattering, light confinement, and control. Additionally, plasmonic nanostructures can trap light within the active layer of a photovoltaic device, increasing the optical path length and improving the overall efficiency of the device. Finally, plasmonic hot carriers generated by plasmonic nanostructures can be used to drive chemical reactions or electrical current, leading to improved energy conversion efficiency and higher device performance.

The use of plasmonic nanostructures in photovoltaics faces several challenges. Plasmonic nanostructures can convert absorbed light into heat, leading to energy loss and reduced overall efficiency. Plasmonic nanostructures can be highly tailored for specific functionality, including being designed to enhance absorption in a specific spectral range, but the range may be somewhat narrow and not match the full range of solar radiation. The fabrication and integration of plasmonic nanostructures into photovoltaic devices can be complex and costly, requiring specialized equipment and processes. Furthermore, plasmonic nanostructures may not be compatible with other materials commonly used in photovoltaics, such as silicon, which can limit their practical use in device fabrication.

Plasmonic nanostructures can help to increase the bandwidth of various applications by providing tunability and flexibility in their optical properties. By controlling the size, shape, and composition of plasmonic nanostructures, it is possible to tailor their resonance and absorption properties across a wide range of frequencies, from ultraviolet to infrared. This tunability makes plasmonic nanostructures attractive for applications such as optical communication, sensing, and imaging, where broadband or multiband operation is desirable. Plasmonic nanostructures can also be designed to support multiple resonances simultaneously, which can further broaden the bandwidth and enhance the sensitivity of these devices. Overall, plasmonic nanostructures offer a promising approach to extending the bandwidth and functionality of various optical applications.

## Figures and Tables

**Table 1:** Examples of plasmonic applications and most common optical processes involved (surface plasmon resonance (SPR), localized surface plasmon resonance (LSPR), plasmon-enhanced absorption (PEA), photoelectric effect, and others).

Application	Section	Optical Processes
Photovoltaics	Section 6	LSPR, PEA, directional scattering (Kerker effect), engineered reflection and/or transmission, photoelectric effect
Light Sources	Section 7	SPR, LSPR, PEA, tip focusing, adiabatic focusing, tip backscattering, light-matter interaction and modification of the local density of optical states (Purcell effect), strong feedback and coupling of gain medium to resonant modes (for discussion of ‘plasmonicity’ see Figure 8e)
Sensors, Biosensors, and Chemical Sensors	Section 8, Section 8.1, and Section 8.2	SPR, LSPR, PEA, SERS, nanostructure-enhanced scattering, absorption, transmission, and/or reflection, chiral response, multipolar resonant coupling and/or interference (bound state in the continuum, Fano resonances, Kerker effect), hyperbolic dispersion, strong coupling in two-dimensional materials, see Figure 9a

## Data Availability

The data presented in the author’s plots are available on request from the author. The data are not publicly available due to technical, resource, and time constraints.

## Abbreviations

The following abbreviations are used in this work:

2D	Two-Dimensional
BIC	Bound State in the Continuum
EELS	Energy Electron Loss Spectroscopy
FoM	Figure of Merit
FWHM	Full Width at Half Maximum
LED	Light-Emitting Diode
LSPR	Localized Surface Plasmon Resonance
PEA	Plasmon-Enhanced Absorption
PIRET	Plasmon-Induced Resonance Energy Transfer
SEM	Scanning Electron Microscope
SERS	Surface-Enhanced Raman Scattering
SHG	Second Harmonic Generation
SPASER	Surface Plasmon Amplification by Stimulated Emission of Radiation
SPP	Surface Plasmon Polariton
SPR	Surface Plasmon Resonance
TMDC	Transition Metal Dichalcogenide
TD-DFT+TB	Time-Dependent Density Functional Theory with Tight Binding

## References

[1] Zayats A.V., Smolyaninov I.I., Maradudin A.A. (2005). Nano-optics of surface plasmon polaritons. Phys. Rep..

[2] Stockman M.I., Kneipp K., Bozhevolnyi S.I., Saha S., Dutta A., Ndukaife J., Kinsey N., Reddy H., Guler U., Shalaev V.M. (2018). Roadmap on plasmonics. J. Opt..

[3] Baumberg J.J., Aizpurua J., Mikkelsen M.H., Smith D.R. (2019). Extreme nanophotonics from ultrathin metallic gaps. Nat. Mater..

[4] Yougbar S., Chou H.-L., Yang C.-H., Krisnawati D.I., Jazidie A., Nuh M., Kuo T.-R. (2021). Facet-dependent gold nanocrystals for effective photothermal killing of bacteria. J. Hazard. Mater..

[5] Duan Q., Liu Y., Chang S., Chen H., Chen J.-H. (2021). Surface Plasmonic Sensors: Sensing Mechanism and Recent Applications. Sensors.

[6] Yougbaré S., Mutalik C., Krisnawati D.I., Kristanto H., Jazidie A., Nuh M., Cheng T.-M., Kuo T.-R. (2020). Nanomaterials for the Photothermal Killing of Bacteria. Nanomaterials.

[7] Novotny L., Hecht B. (2012). Principles of Nano-Optics.

[8] Stockman M.I., Kling M.F., Kleineberg U., Krausz F. (2007). Attosecond Nanoplasmonic Field Microscope. Nat. Photonics.

[9] Xiao S., Mortensen N.A., Jauho A.-P. (2008). Nanostructure design for surface-enhanced Raman spectroscopy—Prospects and limits. J. Eur. Opt. Soc..

[10] Ozturk Z.F., Xiao S., Yan M., Wubs M., Jauho A.-P., Mortensen N.A. (2011). Field enhancement at metallic interfaces due to quantum confinement. J. Nanophoton..

[11] Kneipp K., Kneipp H. (2006). Single-molecule Raman scattering. Appl. Spectrosc..

[12] Oulton R.F., Sorger V.J., Genov D.A., Pile D.F.P., Zhang X. (2009). Plasmon lasers at the deep subwavelength scale. Nature.

[13] Zhou Z.-K., Liu J., Bao Y., Wu L., Png C.E., Wang X.-H., Qiu C.-W. (2019). Quantum plasmonics get applied. Prog. Quantum Electron..

[14] Ciracì C., Hill R.T., Mock J.J., Urzhumov Y., Fernández-Domínguez A.I., Maier S.A., Pendry J.B., Chilkoti A., Smith D.R. (2012). Probing the Ultimate Limits of Plasmonic Enhancement. Science.

[15] Raza S., Stenger N., Kadkhodazadeh S., Fischer S.V., Kostesha N., Jauho A.-P., Burrows A., Wubs M., Mortensen N.A. (2013). Blueshift of the surface plasmon resonance in silver nanoparticles studied with EELS. Nanophotonics.

[16] Scholl J.A., Koh A.L., Dionne J.A. (2012). Quantum plasmon resonances of individual metallic nanoparticles. Nature.

[17] Kongsuwan N., Xiong X., Bai P., You J.-B., Png C.E., Wu L., Hess O. (2019). Quantum Plasmonic Immunoassay Sensing. Nano Lett..

[18] Babicheva V.E., Vergeles S.S., Vorobev P.E., Burger S. (2012). Localized surface plasmon modes in a system of two interacting metallic cylinders. J. Opt. Soc. Am. B.

[19] Babicheva V.E., Nehls J.M., Moloney J.V. Plasmonic Resonances and Light Generation in Nanoparticle Dimers. Proceedings of the 2019 International Applied Computational Electromagnetics Society Symposium (ACES).

[20] Asadi-Aghbolaghi N., Rüger R., Jamshidi Z., Visscher L. (2020). TD-DFT+TB: An Efficient and Fast Approach for Quantum Plasmonic Excitations. J. Phys. Chem. C.

[21] Shalaev V.M., Stockman M.I. (1987). Optical Properties of Fractal Clusters (Susceptibility, Surface-Enhanced Raman Scattering by Impurities). Sov. Phys. JETP.

[22] Butenko A.V., Shalaev V.M., Stockman M.I. (1988). Giant Impurity Nonlinearities in Optics of Fractal Clusters. Sov. Phys. JETP.

[23] Markel V.A., Muratov L.S., Stockman M.I., George T.F. (1991). Theory and Numerical Simulation of Optical Properties of Fractal Clusters. Phys. Rev. B.

[24] Shalaev V.M., Stockman M.I., Botet R. (1992). Resonant Excitations and Nonlinear Optics of Fractals. Phys. A.

[25] Stockman M.I., Shalaev V.M., Moskovits M., Botet R., George T.F. (1992). Enhanced Raman-Scattering by Fractal Clusters–Scale-Invariant Theory. Phys. Rev. B.

[26] Kneipp K., Wang Y., Kneipp H., Perelman L.T., Itzkan I., Dasari R., Feld M.S. (1997). Single Molecule Detection Using Surface-Enhanced Raman Scattering (SERS). Phys. Rev. Lett..

[27] Tsai D.P., Kovacs J., Wang Z., Moskovits M., Shalaev V.M., Suh J.S., Botet R. (1994). Photon Scanning Tunneling Microscopy Images of Optical Excitations of Fractal Metal Colloid Clusters. Phys. Rev. Lett..

[28] Stockman M., George T. (1994). Photon Tunneling Microscope Reveals Local Hot-Spots. Phys. World.

[29] Stockman M.I., Pandey L.N., Muratov L.S., George T.F. (1995). Photon Scanning-Tunneling-Microscopy Images of Optical-Excitations of Fractal Metal Colloid Clusters-Comment. Phys. Rev. Lett..

[30] Stockman M.I., Pandey L.N., George T.F. (1996). Inhomogeneous Localization of Polar Eigenmodes in Fractals. Phys. Rev. B.

[31] Larkin I.A., Stockman M.I., Achermann M., Klimov V.I. (2004). Dipolar Emitters at Nanoscale Proximity of Metal Surfaces: Giant Enhancement of Relaxation in Microscopic Theory. Phys. Rev. B.

[32] Larkin I.A., Stockman M.I. (2005). Imperfect Perfect Lens. Nano Lett..

[33] Aizpurua J., Rivacoba A. (2008). Nonlocal Effects in the Plasmons of Nanowires and Nanocavities Excited by Fast Electron Beams. Phys. Rev. B.

[34] Stockman M.I. (1997). Chaos and Spatial Correlations for Dipolar Eigenproblems. Phys. Rev. Lett..

[35] Stockman M.I., Faleev S.V., Bergman D.J. (2001). Localization Versus Delocalization of Surface Plasmons in Nanosystems: Can One State Have Both Characteristics?. Phys. Rev. Lett..

[36] Stockman M.I., Faleev S.V., Bergman D.J. (2002). Coherent Control of Femtosecond Energy Localization in Nanosystems. Phys. Rev. Lett..

[37] Li K., Stockman M.I., Bergman D.J. (2003). Self-Similar Chain of Metal Nanospheres as an Efficient Nanolens. Phys. Rev. Lett..

[38] Stockman M.I., Faleev S.V., Bergman D.J. (2003). Femtosecond Energy Concentration in Nanosystems: Coherent Control. Phys. B.

[39] Nordlander P., Oubre C., Prodan E., Li K., Stockman M.I. (2004). Plasmon Hybridization in Nanoparticle Dimers. Nano Lett..

[40] Stockman M.I., Bergman D.J., Kobayashi T. (2004). Coherent Control of Nanoscale Localization of Ultrafast Optical Excitation in Nanosystems. Phys. Rev. B.

[41] Stockman M.I., Bergman D.J., Anceau C., Brasselet S., Zyss J. (2004). Enhanced Second-Harmonic Generation by Metal Surfaces with Nanoscale Roughness: Nanoscale Dephasing, Depolarization, and Correlations. Phys. Rev. Lett..

[42] Evlyukhin A.B., Bozhevolnyi S.I. (2015). Resonant unidirectional and elastic scattering of surface plasmon polaritons by high refractive index dielectric nanoparticles. Phys. Rev. B.

[43] Yaroshenko V., Zuev D., Evlyukhin A.B. (2022). Resonant channeling of light near metal surface by passive and active silicon nanoparticles. Surf. Interfaces.

[44] Kinsey N., Ferrera M., Naik G.V., Babicheva V.E., Shalaev V.M., Boltasseva A. (2014). Experimental demonstration of titanium nitride plasmonic interconnects. Opt. Express.

[45] Babicheva V.E., Malureanu R., Lavrinenko A.V. (2013). Plasmonic finite-thickness metal–semiconductor–metal waveguide as ultra-compact modulator. Photonics Nanostruct. Fundam. Appl..

[46] Babicheva V.E., Lavrinenko A.V. (2012). Surface plasmon polariton modulator with optimized active layer. Nanophotonics IV.

[47] Chebykin A.V., Babicheva V.E., Iorsh I.V., Orlov A.A., Belov P.A., Zhukovsky S.V. (2016). Enhancement of the Purcell factor in multiperiodic hyperboliclike metamaterials. Phys. Rev. A.

[48] Babicheva V.E. (2017). Long-range propagation of plasmon and phonon polaritons in hyperbolic-metamaterial waveguides. J. Opt..

[49] Maier S.A. (2007). Plasmonics: Fundamentals and Applications.

[50] Babicheva V.E., Gamage S., Zhen L., Cronin S.B., Yakovlev V.S., Abate Y. (2018). Near-field Surface Waves in Few-Layer MoS2. ACS Photonics.

[51] Abate Y., Gamage S., Zhen L., Cronin S.B., Wang H., Babicheva V., Javani M.H., Stockman M.I. (2016). Nanoscopy reveals surface-metallic black phosphorus. Light. Sci. Appl..

[52] Boulesbaa A., Babicheva V.E., Wang K., Kravchenko I.I., Lin M.W., Briggs D.P., Puretzky A.A., Geohegan D.B., Eres G., Liu J. (2016). Ultrafast dynamics of metal plasmons induced by 2D semiconductor excitons in hybrid nanostructure arrays. ACS Photonics.

[53] Yankovich A.B., Munkhbat B., Baranov D.G., Cuadra J., Olsén E., Lourenço-Martins H., Tizei L.H.G., Kociak M., Olsson E., Shegai T. (2019). Visualizing Spatial Variations of Plasmon–Exciton Polaritons at the Nanoscale Using Electron Microscopy. Nano Lett..

[54] Wang Z., Dong Z., Zhu H., Cao T., Liu X., Song Y., Wang S., Li L., Zhou W., Zhang Y. (2018). Selectively plasmon-enhanced second-harmonic generation from monolayer tungsten diselenide on flexible substrates. ACS Nano.

[55] Hu G., Hong X., Wang K., Gong Y., Yang J., Shi Y., Yang S., Zhang B., Gong Q., Sun X. (2019). Coherent steering of nonlinear chiral valley photons with a synthetic au-ws2 metasurface. Nat. Photonics.

[56] Yan S., Zhu X., Dong J., Ding Y., Xiao S. (2020). 2D materials integrated with metallic nanostructures: Fundamentals and optoelectronic applications. Nanophotonics.

[57] Wood R.W. (1902). On a remarkable case of uneven distribution of light in a diffraction grating spectrum. Philos. Mag..

[58] Wood R.W. (1912). Diffraction Gratings with Controlled Groove form and Abnormal Distribution of intensity. Phil. Mag..

[59] Hessel A., Oliner A.A. (1965). A New Theory of Wood’s Anomalies on Optical Gratings. Appl. Opt..

[60] Evlyukhin A.B., Reinhardt C., Zywietz U., Chichkov B. (2012). Collective resonances in metal nanoparticle arrays with dipole-quadrupole interactions. Phys. Rev. B.

[61] Kravets V.G., Kabashin A.V., Barnes W.L., Grigorenko A.N. (2018). Plasmonic surface lattice resonances: A review of properties and applications. Chem. Rev..

[62] Babicheva V., Staude I., Gérard D. (2019). Collective effects and coupling phenomena in resonant optical metasurfaces: Introduction. J. Opt. Soc. Am. B.

[63] Karimi V., Babicheva V.E. (2023). Dipole-lattice nanoparticle resonances in finite arrays.

[64] Babicheva V.E. (2018). Lattice effect in Mie-resonant dielectric nanoparticle array under the oblique light incidence. MRS Commun..

[65] Evlyukhin A.B., Reinhardt C., Evlyukhin E., Chichkov B.N. (2013). Multipole Analysis of Light Scattering by Arbitrary-Shaped Nanoparticles on a Plane Surface. J. Opt. Soc. Am. B.

[66] Evlyukhin A.B., Chichkov B.N. (2019). Multipole decompositions for directional light scattering. Phys. Rev. B.

[67] Alaee R., Rockstuhl C., Fernandez-Corbaton I. (2019). Exact multipolar decompositions with applications in nanophotonics. Adv. Opt. Mater..

[68] Alaee R., Rockstuhl C., Fernandez-Corbaton I. (2018). An electromagnetic multipole expansion beyond the long-wavelength approximation. Opt. Commun..

[69] Babicheva V.E., Evlyukhin A.B. (2018). Metasurfaces with electric quadrupole and magnetic dipole resonant coupling. ACS Photonics.

[70] Babicheva V.E., Evlyukhin A. (2021). Multipole lattice effects in high refractive index metasurfaces. J. Appl. Phys..

[71] Babicheva V.E., Evlyukhin A.B. (2019). Analytical model of resonant electromagnetic dipole-quadrupole coupling in nanoparticle arrays. Phys. Rev. B.

[72] Babicheva V.E., Evlyukhin A.B. (2018). Interplay and coupling of electric and magnetic multipole resonances in plasmonic nanoparticle lattices. MRS Commun..

[73] Zhao W., Jiang H., Liu B., Jiang Y., Tang C., Li J. (2015). Fano Resonance Based Optical Modulator Reaching 85% Modulation Depth. Appl. Phys. Lett..

[74] Zhao W., Leng X., Jiang Y. (2015). Fano Resonance in All-Dielectric Binary Nanodisk Array Realizing Optical Filter with Efficient Linewidth Tuning. Opt. Express.

[75] Liu Z., Li J., Liu Z., Li W., Li J., Gu C., Yuan Z. (2017). Fano Resonance Rabi Splitting of Surface Plasmons. Sci. Rep..

[76] Karimi V., Babicheva V.E. (2023). Multipole Mie Resonances in MXene-Antenna Arrays.

[77] Han A., Moloney J.V., Babicheva V.E. (2022). Applicability of multipole decomposition to plasmonic- and dielectric-lattice resonances. J. Chem. Phys..

[78] Zhukovsky S.V., Babicheva V.E., Uskov A.V., Protsenko I.E., Lavrinenko A.V. (2014). Enhanced electron photoemission by collective lattice resonances in plasmonic nanoparticle-array photodetectors and solar cells. Plasmonics.

[79] Zhukovsky S.V., Babicheva V.E., Uskov A.V., Protsenko I.E., Lavrinenko A.V. (2014). Electron photoemission in plasmonic nanoparticle arrays: Analysis of collective resonances and embedding effects. Appl. Phys. A.

[80] Bosomtwi D., Osiński M., Babicheva V.E. (2021). Lattice Effect for Enhanced Hot-Electron Generation in Nanoelectrodes. Opt. Mater. Express.

[81] Michaeli L., Keren-Zur S., Avayu O., Suchowski H., Ellenbogen T. (2017). Nonlinear Surface Lattice Resonance in Plasmonic Nanoparticle Arrays. Phys. Rev. Lett..

[82] Han A., Dineen C., Babicheva V.E., Moloney J.V. (2020). Second Harmonic Generation in Metasurfaces with Multipole Resonant Coupling. Nanophotonics.

[83] Han A., Dineen C., Moloney J.V., Babicheva V. Nonlinear Effects in Mie Resonant Plasmonic Lattices. Proceedings of the 2022 IEEE Research and Applications of Photonics in Defense Conference (RAPID).

[84] Beer S., Gour J., Alberucci A., David C., Nolte S., Zeitner U.D. (2022). Second harmonic generation under doubly resonant lattice plasmon excitation. Opt. Express.

[85] Babicheva V.E., Moloney J.V. (2019). Lattice Zenneck modes on subwavelength antennas. Laser Photonics Rev..

[86] Romero A., Islam M.S., Babicheva V. (2023). Multipole Lattice Resonances in Lossy Material. CLEO: 2023, Optica Technical Digest.

[87] Islam S., Babicheva V.E. (2023). Lattice Resonances of Lossy Transition Metal and Metalloid Antennas. MRS Adv..

[88] Babicheva V.E., Moloney J.V. (2019). Lattice Resonances in Transdimensional WS2 Nanoantenna Arrays. Appl. Sci..

[89] Ahmed H., Babicheva V.E. (2020). Nanostructured Tungsten Disulfide WS2 as Mie Scatterers and Nanoantennas. MRS Adv..

[90] Bosomtwi D., Osiński M., Babicheva V.E. Mode Coupling and Rabi Splitting in Transdimensional Photonic Lattices. Proceedings of the 2020 IEEE 20th International Conference on Nanotechnology (IEEE-NANO).

[91] Baryshnikova K., Petrov M., Babicheva V.E., Belov P. (2016). Plasmonic and silicon spherical nanoparticle antireflective coatings. Sci. Rep..

[92] Babicheva V.E., Zhukovsky S.V., Ikhsanov R.S., Protsenko I.E., Smetanin I.V., Uskov A. (2015). Hot Electron Photoemission from Plasmonic Nanostructures: The Role of Surface Photoemission and Transition Absorption. ACS Photonics.

[93] Karimi V., Babicheva V.E. (2023). MXene-Antenna Electrode with Collective Multipole Resonances.

[94] Uskov A.V., Protsenko I.E., Ikhsanov R.S., Babicheva V.E., Zhukovsky S.V., Lavrinenko A.V., OReilly E.P., Xu H. (2014). Internal photoemission from plasmonic nanoparticles: Comparison between surface and volume photoelectric effects. Nanoscale.

[95] Zhukovsky S.V., Babicheva V.E., Evlyukhin A.B., Protsenko I.E., Lavrinenko A.V., Uskov A.V. (2014). Giant photogalvanic effect in noncentrosymmetric plasmonic nanoparticles. Phys. Rev. X.

[96] Zhukovsky S.V., Protsenko I.E., Ikhsanov R.S., Smetanin I.V., Babicheva V.E., Uskov A.V. (2015). Transition absorption as a mechanism of surface photoelectron emission from metals. Phys. Status Solidi-(Rrl)-Rapid Res. Lett..

[97] Ikhsanov R.S., Babicheva V.E., Protsenko I.E., Uskov A.V., Guzhva M.E. (2015). Bulk photoemission from metal films and nanoparticles. Quantum Electron..

[98] Knight M.W., Sobhani H., Nordlander P., Halas N.J. (2011). Photodetection with active optical antennas. Science.

[99] Novitsky A., Uskov A.V., Gritti C., Protsenko I.E., Kardynał B.E., Lavrinenko A.V. (2012). Photon absorption and photocurrent in solar cells below semiconductor bandgap due to electron photoemission from plasmonic nanoantennas. Prog. Photovolt. Res. Appl..

[100] Protsenko I.E., Uskov A.V. (2012). Photoemission from metal nanoparticles. Phys. Usp..

[101] Knight M.W., Wang Y., Urban A.S., Sobhani A., Zheng B.Y., Nordlander P., Halas N.J. (2013). Embedding plasmonic nanostructure diodes enhances hot electron emission. Nano Lett..

[102] Sobhani A., Knight M.W., Wang Y., Zheng B., King N.S., Brown L.V., Fang Z., Nordlander P., Halas N.J. (2013). Narrowband photodetection in the near-infrared with a plasmon-induced hot electron device. Nat. Commun..

[103] Nishijima Y., Ueno K., Yokota Y., Murakoshi K., Misawa H. (2010). Plasmon-Assisted Photocurrent Generation from Visible to Near-Infrared Wavelength Using a Au-Nanorods/TiO2 Electrode. J. Phys. Chem. Lett..

[104] Takahashi Y., Tatsuma T. (2011). Solid State Photovoltaic Cells Based on Localized Surface Plasmon-Induced Charge Separation. Appl. Phys. Lett..

[105] Moulin E.A., Paetzold U.W., Pieters B.E., Reetz W., Carius R. (2013). Plasmon-induced photoexcitation of “hot” electrons and “hot” holes in amorphous silicon photosensitive devices containing silver nanoparticles. J. Appl. Phys..

[106] White T.P., Catchpole K.R. (2012). Plasmon-enhanced internal photoemission for photovoltaics: Theoretical efficiency limits. Appl. Phys. Lett..

[107] Mubeen S., Lee J., Singh N., Krämer S., Stucky G.D., Moskovits M. (2013). An Autonomous Photosynthetic Device in Which All Charge Carriers Derive from Surface Plasmons. Nat. Nanotechnol..

[108] Koppens F.H.L., Chang D.E., Garcia de Abajo F.J. (2011). Graphene Plasmonics: A Platform for Strong Light-Matter Interactions. Nano Lett..

[109] Yang H., Heo J., Park J., Seo J., Kim T.H., Kim K.S., Kim J., Choi S., Shin H.-J., Kang J.-W. (2012). Graphene Barristor, a Triode Device with a Gate-Controlled Schottky Barrier. Science.

[110] Stockman M.I. (2008). Spasers Explained. Nat. Photonics.

[111] Stockman M.I. (2010). The Spaser as a Nanoscale Quantum Generator and Ultrafast Amplifier. J. Opt..

[112] Ma R.M., Oulton R.F., Sorger V.J., Zhang X. (2012). Plasmon Lasers: Coherent Light Source at Molecular Scales. Laser Photonics Rev..

[113] Sidiropoulos T.P.H., Roder R., Geburt S., Hess O., Maier S.A., Ronning C., Oulton R.F. (2014). Ultrafast Plasmonic Nanowire Lasers near the Surface Plasmon Frequency. Nat. Phys..

[114] Stockman M. (2014). Plasmonic Lasers: On the Fast Track. Nat. Phys..

[115] Ning C.Z., Wunner G., Pelster A. (2016). Nanolasers: Current Status of the Trailblazer of Synergetics. Selforganization in Complex Systems: The Past, Present, and Future of Synergetics.

[116] Ning C.-Z. (2021). Spaser or plasmonic nanolaser? - Reminiscences of discussions and arguments with Mark Stockman. Nanophotonics.

[117] Wang S., Chen H.-Z., Ma R.-M. (2018). High Performance Plasmonic Nanolasers with External Quantum Efficiency Exceeding 10%. Nano Lett..

[118] Johnson P.B., Christy R.W. (1972). Optical constants of the noble metals. Phys. Rev. B.

[119] Bergman D.J., Stockman M.I. (2003). Surface Plasmon Amplification by Stimulated Emission of Radiation: Quantum Generation of Coherent Surface Plasmons in Nanosystems. Phys. Rev. Lett..

[120] Noginov M.A., Zhu G., Belgrave A.M., Bakker R., Shalaev V.M., Narimanov E.E., Stout S., Herz E., Suteewong T., Wiesner U. (2009). Demonstration of a Spaser-Based Nanolaser. Nature.

[121] Sorger V.J., Zhang X. (2011). Spotlight on Plasmon Lasers. Science.

[122] Ma R.-M., Oulton R.F., Sorger V.J., Bartal G., Zhang X. (2010). Room-Temperature Sub-Diffraction-Limited Plasmon Laser by Total Internal Reflection. Nat. Mater..

[123] Ma R.-M., Ota S., Li Y., Yang S., Zhang X. (2014). Explosives Detection in a Lasing Plasmon Nanocavity. Nat. Nanotechnol..

[124] Wu C.Y., Kuo C.T., Wang C.Y., He C.L., Lin M.H., Ahn H., Gwo S. (2011). Plasmonic Green Nanolaser Based on a Metal-Oxide-Semiconductor Structure. Nano Lett..

[125] Lu Y.-J., Kim J., Chen H.-Y., Wu C., Dabidian N., Sanders C.E., Wang C.-Y., Lu M.-Y., Li B.-H., Qiu X. (2012). Plasmonic Nanolaser Using Epitaxially Grown Silver Film. Science.

[126] Lu Y.-J., Wang C.-Y., Kim J., Chen H.-Y., Lu M.-Y., Chen Y.-C., Chang W.-H., Chen L.-J., Stockman M.I., Shih C.-K. (2014). All-Color Plasmonic Nanolasers with Ultralow Thresholds: Autotuning Mechanism for Single-Mode Lasing. Nano Lett..

[127] Stockman M.I. (2004). Nanofocusing of Optical Energy in Tapered Plasmonic Waveguides. Phys. Rev. Lett..

[128] Giugni A., Torre B., Toma A., Francardi M., Malerba M., Alabastri A., Proietti Zaccaria R., Stockman M.I., Di Fabrizio E. (2013). Hot-Electron Nanoscopy Using Adiabatic Compression of Surface Plasmons. Nat. Nano.

[129] Zhou W., Dridi M., Suh J.Y., Kim C.H., Co D.T., Wasielewski M.R., Schatz G.C., Odom T.W. (2013). Lasing action in strongly coupled plasmonic nanocavity arrays. Nat. Nano.

[130] Hakala T.K., Rekola H.T., Vakevainen A.I., Martikainen J.-P., Necada M., Moilanen A.J., Tarma P. (2017). Lasing in dark and bright modes of a finite-sized plasmonic lattice. Nat. Commun..

[131] Tittl A., Giessen H., Liu N. (2014). Plasmonic gas and chemical sensing. Nanophotonics.

[132] Hoener B.S., Kirchner S.R., Heiderscheit T.S., Collins S.S.E., Chang W.-S., Link S., Landes C.F. (2018). Plasmonic Sensing and Control of Single-Nanoparticle Electrochemistry. Chem.

[133] Zhu Z., Liu L., Liu Z., Zhang Y., Zhang Y. (2017). Surface-plasmon-resonance-based optical-fiber temperature sensor with high sensitivity and high figure of merit. Opt. Lett..

[134] Yaraki M.T., Tan Y.N. (2020). Metal Nanoparticles-Enhanced Biosensors: Synthesis, Design and Applications in Fluorescence Enhancement and Surface-enhanced Raman Scattering. Chem. Asian J..

[135] Shrivastav A.M., Cvelbar U., Abdulhalim I. (2021). A comprehensive review on plasmonic-based biosensors used in viral diagnostics. Commun. Biol..

[136] Chylek J., Maniakova P., Hlubina P., Sobota J., Pudis D. (2022). Highly Sensitive Plasmonic Structures Utilizing a Silicon Dioxide Overlayer. Nanomaterials.

[137] Offermans P., Schaafsma M.C., Rodriguez S.R.K., Zhang Y., Crego-Calama M., Brongersma S.H., Gómez Rivas J. (2011). Universal scaling of the figure of merit of plasmonic sensors. ACS Nano.

[138] Qin J., Jiang S., Wang Z., Cheng X., Li B., Shi Y., Tsai D.P., Liu A.Q., Huang W., Zhu W. (2022). Metasurface Micro/Nano-Optical Sensors: Principles and Applications. ACS Nano.

[139] Zhao W., Ju D., Jiang Y. (2015). Sharp Fano Resonance within Bi-periodic Silver Particle Array and Its Application as Plasmonic Sensor with Ultra-high Figure of Merit. Plasmonics.

[140] Bosomtwi D., Babicheva V.E. (2023). Beyond Conventional Sensing: Hybrid Plasmonic Metasurfaces and Bound States in the Continuum. Nanomaterials.

[141] Mejía-Salazar J.R., Oliveira O. (2018). N. Plasmonic Biosensing Focus Review. Chem. Rev..

[142] Mauriz E. (2020). Recent Progress in Plasmonic Biosensing Schemes for Virus Detection. Sensors.

[143] Balbinot S., Srivastav A.M., Vidic J., Abdulhalim I., Manzano M. (2021). Plasmonic biosensors for food control. Trends Food Sci. Technol..

[144] Song L., Chen J., Xu B.B., Huang Y. (2021). Flexible Plasmonic Biosensors for Healthcare Monitoring: Progress and Prospects. ACS Nano.

[145] Liu Y., Zhang X. (2021). Microfluidics-Based Plasmonic Biosensing System Based on Patterned Plasmonic Nanostructure Arrays. Micromachines.

[146] Mauriz E., Lechuga L.M. (2021). Plasmonic Biosensors for Single-Molecule Biomedical Analysis. Biosensors.

[147] Carcelen M., Vidal V., Franco A., Gomez M., Moreno F., Fernandez-Luna J.L. (2022). Plasmonic Biosensing for Label-Free Detection of Two Hallmarks of Cancer Cells: Cell-Matrix Interaction and Cell Division. Biosensors.

[148] Park J.A., Amri C., Kwon Y., Lee J.-H., Lee T. (2022). Recent Advances in DNA Nanotechnology for Plasmonic Biosensor Construction. Biosensors.

[149] Ferrari E. (2023). Gold Nanoparticle-Based Plasmonic Biosensors. Biosensors.

[150] Zhang R., Zhang Z., Fan Y., Zhang H., Chu J. (2023). Single-Layer Transmissive Chiral Plasma Metasurface with High Circular Polarization Extinction Ratio in Visible Wavelength. Nanomaterials.

[151] Martínez-Hernández M.E., Rivero P.J., Goicoechea J., Arregui F.J. (2021). Trends in the Implementation of Advanced Plasmonic Materials in Optical Fiber Sensors (2010–2020). Chemosensors.

[152] Arcadio F., Zeni L., Perri C., D’Agostino G., Chiaretti G., Porto G., Minardo A., Cennamo N. (2021). Bovine Serum Albumin Protein Detection by a Removable SPR Chip Combined with a Specific MIP Receptor. Chemosensors.

[153] Khattak A., Wei L. (2022). Fano Resonance Hybrid Waveguide-Coupled Plasmonic Sensor Using Transparent Conductive Oxide in the Near-Infrared Range. Photonics.

[154] Wang H., Fang T., Liu H., Wei T., Dai Z. (2022). Gold Nanostar-Based Sensitive Catechol Plasmonic Colorimetric Sensing Platform with Ultra-Wide Detection Range. Chemosensors.

